# Cerebral multimodality monitoring in adult neurocritical care patients with acute brain injury: A narrative review

**DOI:** 10.3389/fphys.2022.1071161

**Published:** 2022-12-01

**Authors:** Jeanette Tas, Marek Czosnyka, Iwan C. C. van der Horst, Soojin Park, Caroline van Heugten, Mypinder Sekhon, Chiara Robba, David K. Menon, Frederick A. Zeiler, Marcel J. H. Aries

**Affiliations:** ^1^ Maastricht University Medical Center +, Department of Intensive Care Medicine, Maastricht University, Maastricht, Netherlands; ^2^ School for Mental Health and Neuroscience (MHeNS), Maastricht University, Maastricht, Netherlands; ^3^ Brain Physics Laboratory, Department of Clinical Neurosciences, Division of Neurosurgery, University of Cambridge, Cambridge, United Kingdom; ^4^ Cardiovascular Research Institute Maastricht (CARIM), Maastricht, Netherlands; ^5^ Departments of Neurology and Biomedical Informatics, Columbia University, New York, NY, United States; ^6^ Department of Neuropsychology and Psychopharmacology, Faculty of Psychology and Neuroscience, Maastricht University, Maastricht, Netherlands; ^7^ Division of Critical Care Medicine, Department of Medicine, University of British Columbia, Vancouver, BC, Canada; ^8^ Djavad Mowafaghian Centre for Brain Health, University of British Columbia, Vancouver, BC, Canada; ^9^ Department of Anaesthesia and Intensive Care, Policlinico Santino IRCCS for Oncology and Neuroscience, Dipartimento di Scienze Chirurgiche Diagnostiche Integrate, University of Genova, Genova, Italy; ^10^ University Division of Anaesthesia, Addenbrooke’s Hospital, University of Cambridge, Cambridge, United Kingdom; ^11^ Department of Biomedical Engineering, Faculty of Engineering, University of Manitoba, Winnipeg, MB, Canada; ^12^ Section of Neurosurgery, Department of Surgery, Rady Faculty of Health Sciences, University of Manitoba, Winnipeg, MB, Canada; ^13^ Department of Human Anatomy and Cell Science, Rady Faculty of Health Sciences, University of Manitoba, Winnipeg, MB, Canada; ^14^ Centre on Aging, University of Manitoba, Winnipeg, MB, Canada; ^15^ Department of Clinical Neuroscience, Karolinska Institute, Stockholm, Sweden

**Keywords:** cerebral multimodality monitoring, intensive care, outcome, TBI, SAH, ICH, AIS, HIBI

## Abstract

Cerebral multimodality monitoring (MMM) is, even with a general lack of Class I evidence, increasingly recognized as a tool to support clinical decision-making in the neuroscience intensive care unit (NICU). However, literature and guidelines have focused on unimodal signals in a specific form of acute brain injury. Integrating unimodal signals in multiple signal monitoring is the next step for clinical studies and patient care. As such, we aimed to investigate the recent application of MMM in studies of adult patients with traumatic brain injury (TBI), subarachnoid hemorrhage (SAH), intracerebral hemorrhage (ICH), acute ischemic stroke (AIS), and hypoxic ischemic brain injury following cardiac arrest (HIBI). We identified continuous or daily updated monitoring modalities and summarized the monitoring setting, study setting, and clinical characteristics. In addition, we discussed clinical outcome in intervention studies. We identified 112 MMM studies, including 11 modalities, over the last 7 years (2015–2022). Fifty-eight studies (52%) applied only two modalities. Most frequently combined were ICP monitoring (92 studies (82%)) together with PbtO_2_ (63 studies (56%). Most studies included patients with TBI (59 studies) or SAH (53 studies). The enrollment period of 34 studies (30%) took more than 5 years, whereas the median sample size was only 36 patients (q1- q3, 20–74). We classified studies as either observational (68 studies) or interventional (44 studies). The interventions were subclassified as systemic (24 studies), cerebral (10 studies), and interventions guided by MMM (11 studies). We identified 20 different systemic or cerebral interventions. Nine (9/11, 82%) of the MMM-guided studies included clinical outcome as an endpoint. In 78% (7/9) of these MMM-guided intervention studies, a significant improvement in outcome was demonstrated in favor of interventions guided by MMM. Clinical outcome may be improved with interventions guided by MMM. This strengthens the belief in this application, but further interdisciplinary collaborations are needed to overcome the heterogeneity, as illustrated in the present review. Future research should focus on increasing sample sizes, improved data collection, refining definitions of secondary injuries, and standardized interventions. Only then can we proceed with complex outcome studies with MMM-guided treatment.

## 1 Introduction

Neuromonitoring is used to guide treatment in patients with acute brain injuries. Most neuroscience intensive care units (NICU) in high-income countries have intracranial pressure (ICP) and cerebral perfusion pressure (CPP), along with transcranial Doppler (TCD) and surface electroencephalography (sEEG) as brain monitoring tools available in a selection of their acute brain injured patients (Le Roux et al., 2014; Hutchinson et al., 2015; Carney et al., 2017; Cnossen et al., 2017). Partial pressure of brain tissue oxygenation (PbtO_2_), cerebral temperature (Cerebral T), regional cerebral blood flow (rCBF), jugular bulb venous oximetry (SvjO_2_), cerebral microdialysis (CMD), near-infrared spectroscopy (NIRS) and electrocorticography (ECoG; from invasive electrodes on the cerebral surface) and depth electroencephalography (dEEG) are the other frequently applied modalities (Le Roux et al., 2014; Stocchetti et al., 2017).

Cerebral multimodality monitoring (MMM) is often mentioned in NICU reviews (Makarenko et al., 2016; Stocchetti et al., 2017; Tasneem et al., 2017; Smith, 2018; Al-Mufti et al., 2019; Veldeman et al., 2020a; Yang, 2020), but reviews and guidelines mainly discuss the results of unimodal signals (Le Roux et al., 2014; Carney et al., 2017). The practical application of “combining modalities” is limited by the high-dimensionality of signals and non-standardized methods to present the information at the bedside. Also, clinical context, including imaging results, is not incorporated (Tasneem et al., 2017; Smith, 2018; Al-Mufti et al., 2019; Veldeman et al., 2020a; Yang, 2020). In 2014, Le Roux et al. (2014) formulated five-year expectations and recommendations regarding MMM in acute brain injured patients. They expected patient-specific rather than population-specific thresholds, TCD-based non-invasive measures for ICP monitoring, and advances in the detection of cortical spreading depolarization.

Since the projections by Le Roux et al. were put forward, no overview of the application of MMM studies has been published (Le Roux et al., 2014). However, rigorous insight into MMM of recent years could detect benefits, pitfalls, and gaps for improving future clinical study designs. In this narrative review, we, therefore, aim to investigate the recent applications of cerebral MMM in studies for acute brain injured patients (i.e., adult patients with traumatic brain injury (TBI), subarachnoid hemorrhage (SAH), intracerebral hemorrhage (ICH), acute ischemic stroke (AIS) or hypoxic ischemic brain injury following cardiac arrest (HIBI). Our objectives are (I) to identify which combinations of monitoring modalities are currently applied, in general, and across the different acute brain injuries, (II) to summarize the monitoring setting, study setting, and clinical characteristics, and (III) to discuss the potential added value of MMM on clinical outcome in intervention studies.

## 2 Methods

We identified studies describing combinations of cerebral monitors providing data that updates continuously or on a regular daily basis (i.e. regularly over the day) through a PubMed literature search. We used a stepwise approach for the literature search and identification of eligible studies.

Step 1, for each cerebral monitoring modality, a single PubMed query was used (Supplementary Table S1).
Step 2, each MMM combination (ICP and NIRS, ICP and sEEG, NIRS and TCD, etc.) was used in the search in combination with the general inclusion criteria. The general inclusion criteria were: clinical study, adult (age, >18 years old) patients, article written in English, and an Epub publication period covering Jan 1, 2015 to Jul 1, 2022. These general criteria were selected in the PubMed filters.
Step 3, the abstracts (and, if needed, the full-text studies) were screened for further eligibility: (I) the study had to concern critical care patients with (II) a minimum of five patients and (III) diagnosed with TBI, SAH, ICH, AIS or HIBI.
Step 4, all selected full-text studies were read, and their references were screened for additional studies. The abstracts were read when the reference was used in a MMM context in the main text or when in a reference MMM was part of the title. In addition, the citations of the selected studies were screened in the Web of Science Core collection database (August 2022).
Step 5, we selected MMM studies for which the study aim or objective(s) were related to MMM. We defined MMM application as (I) the application and reporting results of at least two modalities, i.e., modalities that were part of the research protocol, and (II) without aiming to evaluate superiority/inferiority between modalities (validation studies), as these studies are not designed to integrate multiple signals but aim for the (potential) replacement of a signal.
Step 6, we collected the monitoring setting, study setting, and clinical characteristics from each study. In addition, we collected defined secondary injuries from observational and interventional studies. These secondary injuries are the defined cerebral, potential reversible, pathophysiological conditions diagnosed by monitoring, imaging, or other clinical diagnostics. The interventions and the clinical outcome were also collected for the interventional studies. Detailed definitions/descriptions are given in Supplementary Table S2. The collected information resulted in a comprehensive table to support the objectives of our MMM review.

For objective I, we described the number and combinations of the different modalities. The number of monitoring combinations was calculated, and their synergy was visualized in a Circos plot (Krzywinski et al., 2009).

For objective II, we summarized the monitoring setting, study setting, and clinical characteristics of the selected studies between the diseases and reported the results as frequencies or medians (together with interquartile range, q1-q3). Furthermore, we described the secondary injuries studied in observational and interventional studies. Finally, we summarized the interventions that were applied in the MMM studies.

For objective III, we discussed the added value of MMM on clinical outcome in intervention studies.

## 3 Results: Study selection

After the abstract, references, and citation identification, 209 full-text studies were read. From these, 97 studies whose aim or objective(s) were not related to MMM were excluded. These excluded studies were predominantly (52 studies) validation (superiority/inferiority) studies comparing non-invasive TCD-based ICP with invasive ICP monitoring (25 studies). Supplementary Table S3 lists the modalities used for validation. The study selection flowchart is shown in Supplementary Figure S1. In addition, the number of included studies by year can be found in Supplementary Figure S2. For the final analysis, 112 MMM studies were available, of which 59 concerned TBI (53%), 53 SAH (47%), 13 ICH (12%), 5 AIS (4.5%), and 9 HIBI (8%).

## 4 Results objective I and II: Cerebral multimodality monitoring combinations and monitoring setting

**FIGURE 1:**
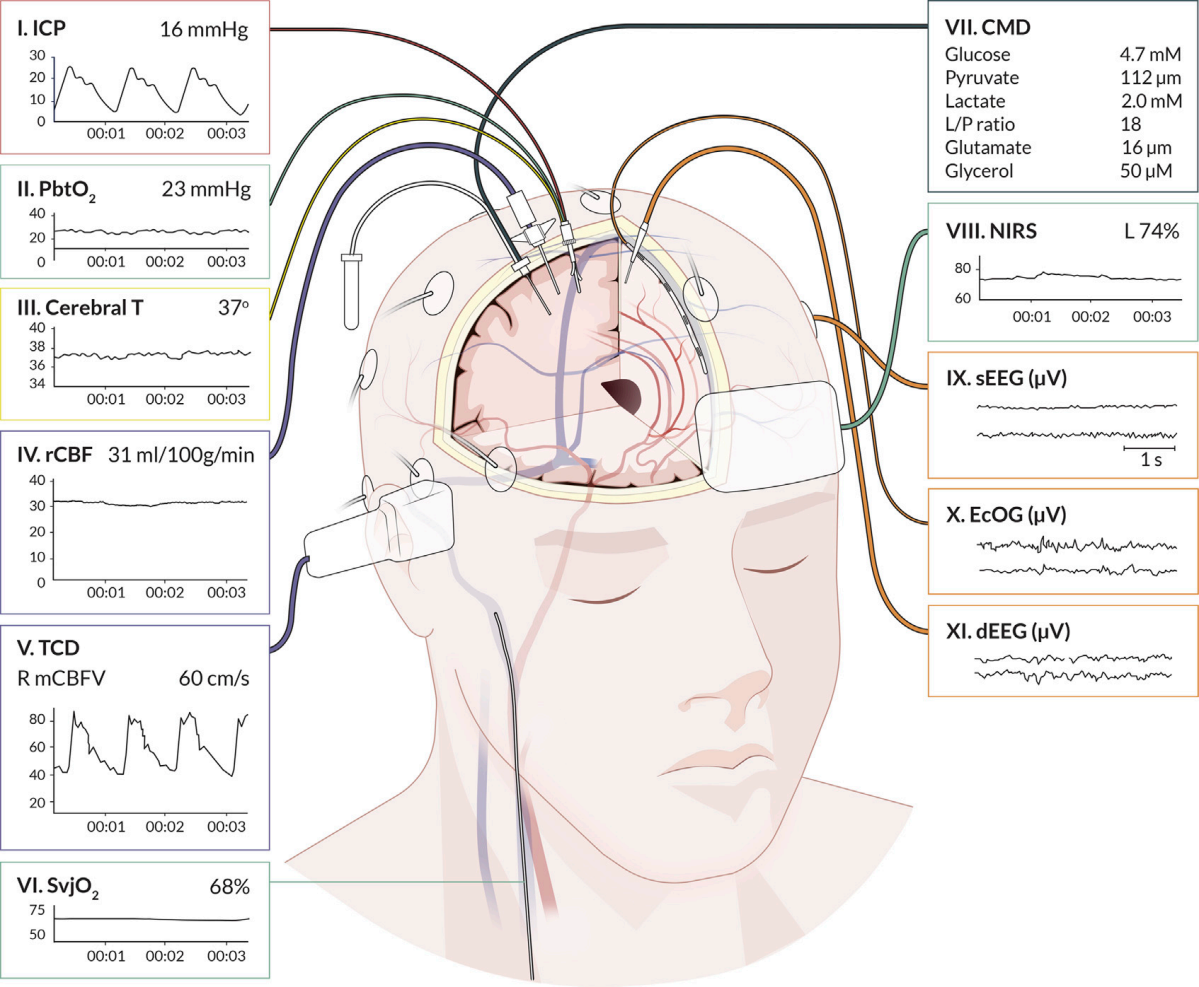
Graphical representation of cerebral multimodality monitoring modalities. The eleven applied monitoring modalities with numbers and (raw) signals. Each modality presents the standard visualization on the bedside monitoring screen. For the readability of the figure, only two neuronal activity monitoring electrodes are displayed. In common practice, the numbers for sEEG are application of 21 electrodes, for ECoG and dEEG 4-8 electrodes. Cerebral T, cerebral temperature; CMD, cerebral microdialysis; dEEG, depth electroencephalography; ECoG, electrocorticography; ICP, intracranial pressure; NIRS, near-infrared spectroscopy; PbtO_2_, partial pressure of brain tissue oxygenation; rCBF, regional cerebral blood flow; sEEG, surface electroencephalography; SvjO_2_, jugular bulb venous oximetry; TCD, transcranial Doppler. Professional illustration by Anna Sieben (Sieben Medical Art).

**FIGURE 2:**
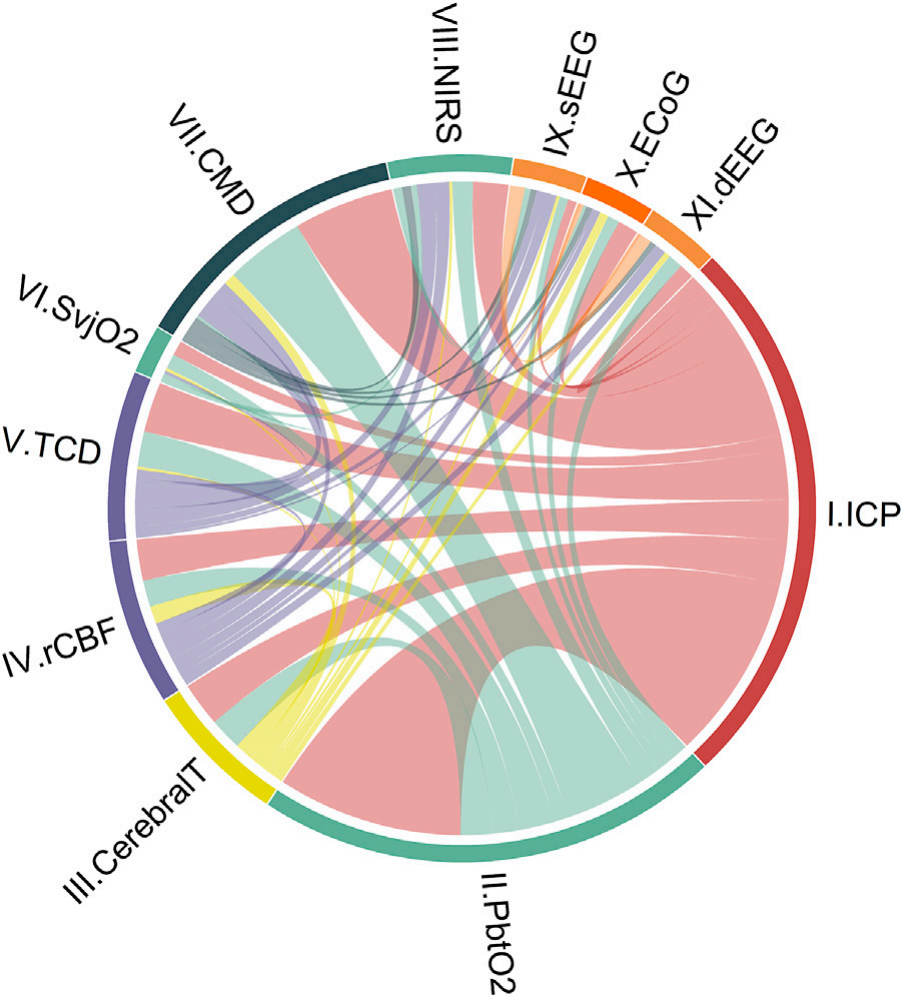
Combinations of cerebral unimodal monitoring modalities in the literature over the last 7 years (112 studies). Circos-plot visualizing connections between unimodal continuous cerebral monitoring modalities. ICP monitoring is the modality most combined, followed by PbtO_2_. As an illustration to understand the distribution of each part: ICP monitoring appears in study 1 in combination with modalities II and III, and in study 2, ICP appears with modalities IV and V. ICP monitoring is then displayed on 2/6 of the circle (ICP + ICP + II + III + IV + V, 6 of which 2x ICP). The colors represent intracranial volume (red), cerebral oxygenation (green), regional cerebral blood flow (purple), cerebral metabolism (dark blue), neuronal electrical activity (orange), and cerebral temperature (yellow). Cerebral T, cerebral temperature; CMD, cerebral microdialysis; dEEG, depth electroencephalography; ECoG, electrocorticography; ICP, intracranial pressure; NIRS, near-infrared spectroscopy; PbtO_2_, partial pressure of brain tissue oxygenation; rCBF, regional cerebral blood flow; sEEG, surface electroencephalography; SvjO_2_, jugular bulb venous oximetry; TCD, transcranial Doppler.

**FIGURE 3:**
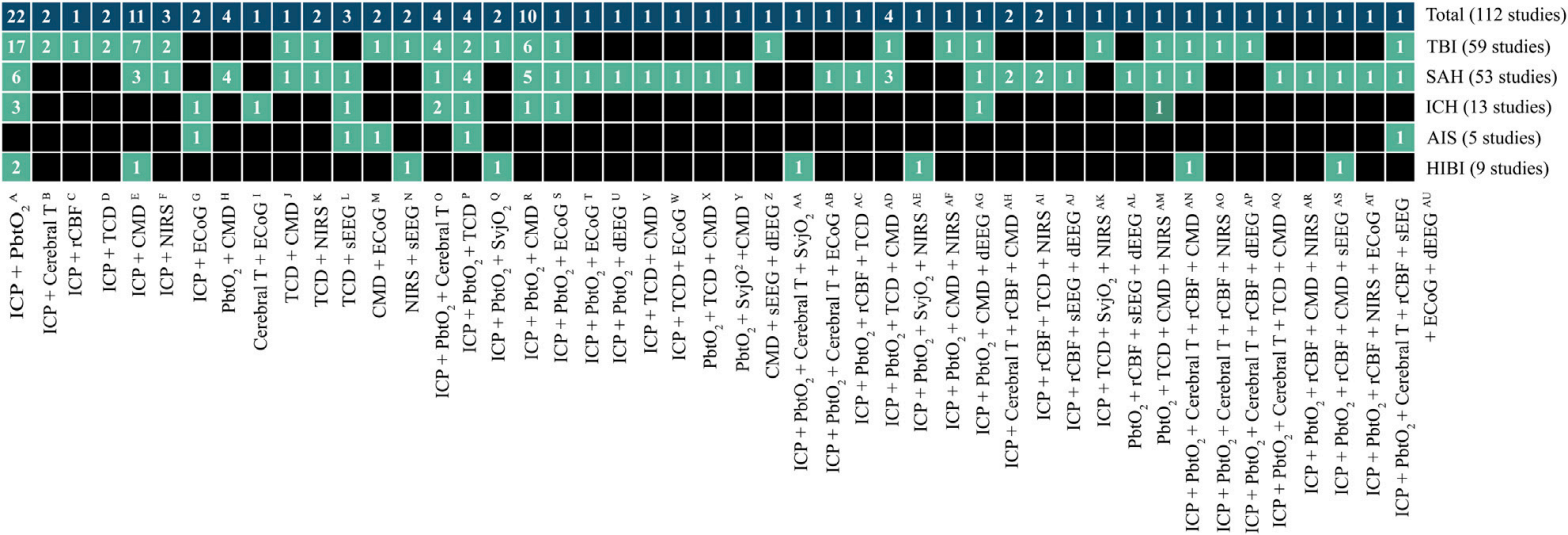
Unique cerebral multimodality monitoring combinations. The 47 unique combinations of MMM are shown. The first upper row shows the total number of studies per combination. The second to the sixth row shows the number of studies per acute brain injury: TBI, SAH, ICH, AIS, and HIBI. Each box describes the number of studies. The boxes in black do not include a monitoring combination for a particular disease. The references of the studies are added to Supplementary Tables S5A–D. The reference numbers per unique combination are: A (11,14,24,33,43,46,47,48,49,53, 69,71,87,90,91,94,97, 104,105,106,107,110); B (51,55); C (4); D (76,31); E (9,38,45,61,62,63,66,67,77,82,101); F (32,37,73); G (8,60); H (17,70,95,98); I (21); J (78); K (6,74); L (23,57,58); M(10,20); N (68,79); O (30,40,42,100); *p* (83,86,93,96); Q (35,44); R (1,3,13,19,34,52,56,59,89,102); S (2); T (27); U (29); V (112); W (64); X (99); Y (5); Z (15); AA (111); AB (16); AC (103); AD (80,92,108,109); AE (81); AF (41); AG (50); AH (12,39); AI (75,88); AJ (18); AK (84); AL (22); AM(72); AN (36); AO (7); AP (26); AQ (85); AR (54); AS (28); AT (65); AU (25). Note: the sum of studies for the individual diseases not count towards the total number of studies because a study can include patients with different diseases. AIS, acute ischemic stroke; Cerebral T, cerebral temperature; CMD, cerebral microdialysis; dEEG, depth electroencephalography; ECoG, electrocorticography; HIBI, hypoxic-ischemic brain injury following cardiac arrest; ICH, intracerebral hemorrhage; ICP, intracranial pressure; NIRS, near-infrared spectroscopy; PbtO_2_, partial pressure of brain tissue oxygenation; rCBF, regional cerebral blood flow; SAH, subarachnoid hemorrhage; sEEG, surface electroencephalography; SvjO_2_, jugular bulb venous oximetry; TBI, traumatic brain injury; TCD, transcranial Doppler.

We identified 11 monitoring modalities that update continuously or on a regular daily basis. The anatomical locations are graphically presented in Figure 1, showing eight invasive (ICP, PbtO_2_, Cerebral T, rCBF, SvjO_2_, CMD, ECoG, dEEG) and three non-invasive (TCD, NIRS, sEEG) modalities. The synergy of the combinations is shown in Figure 2. The individual modalities were integrated into 47 unique combinations (Figure 3). In 58 studies (52%), two modalities were applied, three in 28 studies (25%), and only 26 studies (23%) utilized more than three modalities (Supplementary Figure S3). ICP monitoring was the most frequently combined modality, in 92 studies (82%), with the highest number in TBI patients (53 studies, 90%). The second most applied modality was PbtO_2_ in 71 studies (63%). SvjO_2_ monitoring was only applied in six studies (5.4%) and mainly combined with ICP (5 studies) and PbtO_2_ (5 studies) monitoring. Invasive neuronal activity monitoring (ECoG and dEEG studies, 17 studies) was more common than non-invasive neuronal activity monitoring (sEEG, 10 studies). Regarding non-invasive modalities, TCD was most often studied (25 studies), predominantly in patients with SAH, ICH, and AIS. TCD was not studied in HIBI patients. We studied only modalities that were part of the research protocol. However, 21 SAH studies also mentioned other modalities (mainly ICP, Cerebral T, and TCD), which were only part of the clinical protocol. These modalities were not considered as often only limited, or no continuous information was provided. Supplementary Table S5 lists these modalities for the individual studies. Lastly, only 58% of the studies analyzed more than 24 h of data per patient. A summary of the monitoring settings is given in Table 1 and Supplementary Table S4A.

**TABLE 1:** Monitoring setting of cerebral multimodality monitoring studies (112 studies).

	TBI^a^	SAH^a^	ICH^a^	AIS^a^	HIBI^a^
	59 studies	53 studies	13 studies	5 studies	9 studies
Unimodal modalities, no. of studies (%)					
I. ICP	53 (90)	42 (79)	10 (77)	3 (60)	8 (89)
II. PbtO_2_	39 (66)	39 (74)	10 (77)	2 (40)	7 (78)
III. Cerebral T	10 (17)	7 (13)	3 (23)	1 (20)	2 (22)
IV. rCBF	5 (8.5)	12 (23)	0	1 (20)	2 (22)
V. TCD	9 (15)	18 (34)	3 (23)	2 (40)	0
VI. SvjO_2_	2 (3.4)	1 (1.9)	0	0	3 (33)
VII. CMD	21 (36)	27 (51)	3 (23)	1 (20)	3 (33)
VIII. NIRS	9 (15)	8 (15)	2 (15)	0	2 (22)
IX. sEEG	3 (5.1)	5 (9.4)	1 (7.7)	2 (40)	2 (22)
X. ECoG	2 (3.4)	5 (9.4)	2 (15)	3 (60)	0
XI. dEEG	4 (6.8)	5 (9.4)	1 (7.7)	1 (20)	0
Other neuromonitoring applied (not related to the research protocol), no. of studies (%)					
One modality	9 (15)	21 (40)	3 (23)	1 (20)	1 (11)
Two other modalities	2 (3.4)	4 (7.5)	1 (7.7)	0	0
Duration monitoring used for data analysis, no. of studies (%)					
0–1 hour	8 (14)	4 (7.5)	3 (23)	2 (40)	1 (11)
2–12 hours	8 (14)	8 (15)	2 (15)	0	0
13–23 hours	2 (3.4)	4 (7.5)	0	1 (20)	0
≥24 hours	31 (53)	29 (55)	6 (46)	0	7 (78)
Not reported	10 (17)	8 (15)	2 (15)	2 (40)	1 (11)
ABP zeroing (when ICP monitoring was applied), no. of studies (%)	53 (90)	53 (79)	10 (77)	3 (60)	8 (89)
Heart	9 (17)	7 (17)	2 (20)	0	1 (13)
Foramen of Monro	5 (9.4)	3 (7.1)	3 (30)	0	0
Both	1 (1.9)	1 (2.4)	1 (10)	0	0
Not reported	38 (72)	31 (74)	4 (40)	3 (100)	7 (88)

^a^ Multiple diseases: several studies report more than one disease. These studies are represented for each diagnosis. The percentages are reported as whole numbers. The percentages not count to 100% due to rounding. Definitions are listed in Supplementary Table S2.

ABP, arterial blood pressure; AIS, acute ischemic stroke; HIBI, hypoxic ischemic brain injury; Cerebral T, cerebral temperature; CMD, cerebral microdialysis; dEEG, depth electroencephalography; ECoG, electrocorticography; MMM, multimodality monitoring; ICH, intracerebral hemorrhage; ICP, intracranial pressure; NIRS, near-infrared spectroscopy; No., number; PbtO_2_, partial pressure of brain tissue oxygenation; rCBF, regional cerebral blood flow; SAH, subarachnoid hemorrhage; sEEG, surface electroencephalography; SvjO_2_, jugular bulb venous oximetry; TBI, traumatic brain injury; TCD, transcranial Doppler.

## 5 Results: Objective II study setting and clinical characteristics

The study setting and clinical characteristics are summarized in Table 2 and Supplementary Table S4B. Most were single-center studies (90 studies, 80%) with a median sample size of 36 (q1-q3, 20–74) patients. In 34 studies (30%), patients were enrolled over a period of more than 5 years. TBI studies included more patients compared to SAH studies (TBI 43 (22–100) patients versus SAH 26 (17–69) patients). In addition, TBI studies more often had a multicenter design (TBI, 37% versus SAH, 15%).

**TABLE 2:** Study setting, and clinical characteristics of cerebral multimodality monitoring studies (112 studies).

	TBI^a^	SAH^a^	ICH^a^	AIS^a^	HIBI^a^
	59 studies	53 studies	13 studies	5 studies	9 studies
Multicentre studies, no. of studies (%)	22 (37)	8 (15)	3 (23)	3 (60)	3 (33)
Study enrollment period, no. of studies (%)					
0–1 year	11 (19)	9 (17)	3 (23)	1 (20)	2 (22)
2–3 years	13 (22)	9 (17)	4 (31)	1 (20)	6 (67)
4–5 years	7 (12)	10 (19)	1 (7.7)	1 (20)	0
≥6 years	18 (31)	16 (30)	3 (23)	1 (20)	1 (11)
Not reported	10 (17)	9 (17)	2 (15)	1 (20)	0
Sample sizes, median (q1 – q3)	43 (22–100)	26 (17–69)	47 (25–69)	23 (18–59)	18 (11–65)
Sex, male (%), median (q1 – q3)	75 (60–81)	31 (24–50)	53 (49–60)	39 (20–60)	61 (33–70)
Age range, no. of studies (%)					
18–29 years	1 (1.7)	0	0	0	0
30–39 years	20 (34)	4 (7.5)	1 (7.7)	1 (20)	0
40–49 years	21 (36)	7 (13)	3 (23)	0	6 (67)
50–59 years	14 (24)	37 (70)	5 (38)	4 (80)	2 (22)
60–69 years	0	3 (5.7)	4 (31)	0	1 (11)
Not reported	3 (5.1)	2 (3.8)	0	0	0
Multiple pre-defined diseases per study, no. of studies (%)	16 (27)	15 (28)	9 (69)	2 (40)	3 (33)

^a^ Multiple diseases: some studies report more than one disease. These studies are represented for each diagnosis. Supplementary Tables S5A–D lists the studies.

The percentages are reported as whole numbers. The percentages not count to 100% due to rounding. Definitions are listed in Supplementary Table S2

AIS, acute ischemic stroke; HIBI, hypoxic-ischemic brain injury following cardiac arrest; ICH, intracerebral hemorrhage; No., number; MMM, multimodality monitoring; SAH, subarachnoid hemorrhage; TBI, traumatic brain injury; q1-q3, interquartile range

Eighteen studies (16%) included combinations of acute brain injured patients. Especially, ICH and AIS were combined with other acute brain injuries. There were only four single disease studies of ICH and only three of AIS. Although HIBI is the least contributing group, relatively more single disease studies were included (5 studies) compared to ICH and AIS.

Clinical characteristics differed between diseases. TBI studies included relatively younger male patients (71%<50 years, 75% male), whereas SAH studies included older female (70% 50–59 years, 31% male) patients. HIBI studies included middle-aged, slightly more male patients (67%, 40–49 years, 61% male). Studies that included ICH patients included a wide range of ages (40–69 years, 53% male). AIS included predominantly patients within the range 50–59 years and female (39% male).

## 6 Results objective II: Secondary injuries

Secondary brain injuries are heterogeneous in presentation, with a complex interplay between impairments in diffusion, perfusion, metabolic derangements, and neuronal damage. We studied the different conditions and phenomena defined by the authors of the observational (68 studies) and interventional (44 studies) studies. Authors reported hypo-/hyper perfusion, cerebrovascular autoregulation impairment, ICP plateau waves, spreading depolarization, diffuse cerebral ischemia, vasospasm, and metabolic distress. Due to the inconsistencies in definitions and nomenclature of (single) modalities, no detailed group results across the diseases are presented, but examples are given to explain these inconsistencies.

Authors either allocated patients with/without a specific secondary brain injury and compared differences in MMM signals between the groups, or authors selected a whole group of a particular disease. Then, they reported the secondary brain injuries based on the thresholds of each modality.

In general, the number of secondary brain injuries is large because each modality has its own threshold for impairment, or a combination of modalities defines an impairment. In other words, the definitions of secondary brain injuries are limited by the number of available modalities. For example, Lindner et al. (2021) defined mitochondrial dysfunction (single modality) as: CMD lactate/pyruvate (L/P)-ratio ≥ 40 + CMD-pyruvate ≥ 70 μmol/L, whereas Khellaf et al. defined mitochondrial dysfunction (three modalities) as: CMD L/P-ratio>25 for more than 2 h, ICP <20 mmHg; PbtO_2_ <15 mmHg; PRx <0.3; brain extracellular glucose >1 mmol/L (Khellaf et al., 2022).

In addition, there were inconsistencies in nomenclature for impairments using single modalities. For example, Hosmann et al. (2022) define indications for cerebral ischemia as CMD L/P-ratio >40 CMD-glycerol >100 μmol/L, CMD-lactate >4 mmol/L, whereas Nyholm et al. (2017) defined cerebral ischemia as CMD-L/P ratio >40 and CMD-pyruvate <50 mol/L). For brain tissue hypoxia monitored by PbtO_2_ there were in general two definitions used: PbtO_2_ <15 mmHg (Burnol et al., 2021; Hosmann et al., 2021) or <20 mmHg (Le Roux et al., 2014; Gagnon et al., 2020; Sekhon et al., 2020; Gouvea Bogossian et al., 2021).

## 7 Results objective III: Interventions, potential therapies

We identified systemic- (24 studies), cerebral (10 studies) interventions, and interventions guided by MMM (11 studies). Table 3 and Supplementary Table S4C summarize the study classifications. In addition, one study was classified as MMM-guided and a cerebral intervention.

**TABLE 3:** Study classification of cerebral multimodality monitoring studies (112 studies).

	TBI^a^	SAH^a^	ICH^a^	AIS^a^	HIBI^a^
	59 studies	53 studies	13 studies	5 studies	9 studies
No. of studies (%)
Observational	36 (61)	28 (53)	9 (69)	4 (80)	6 (67)
Systemic intervention	15 (25)	14 (26)	4 (31)	1 (20)	2 (22)
Cerebral intervention	5 (8.5)^b^	5 (9.4)	0	0	0
Interventions guided by MMM	4 (6.8)^b^	6 (11)	0	0	1 (11)
Intervention studies - Clinical outcome endpoint	7 (30)	9 (36)	1 (25)	0	3 (100)
Safety endpoint	8 (14)	10 (19)	2 (15)	1 (20)	2 (22)

^a^ Multiple diseases: several studies report more than one disease. These studies are represented for each diagnosis. Supplementary Tables S5A–D lists the studies.

^b^ One study was classified as both interventions guided by MMM and cerebral intervention (Khellaf et al., 2022).

The percentages are reported as whole numbers. The percentages not count to 100% due to rounding. Definitions are listed in Supplementary Table S2

AIS, acute ischemic stroke; HIBI, hypoxic-ischemic brain injury following cardiac arrest; ICH, intracerebral hemorrhage; MMM, multimodality monitoring; No., number; SAH, subarachnoid hemorrhage; TBI, traumatic brain injury

A total number of 20 different systemic- or cerebral interventions were applied. An example of a systemic intervention is the administration of red blood cell (RBC) transfusion (Sekhon et al., 2015; Kurtz et al., 2016; McCredie et al., 2017; Gouvêa Bogossian et al., 2022). An example of a cerebral intervention is the application of prostacyclin with a beneficial effect on neuronal cell membrane destruction (Koskinen et al., 2019). Examples of MMM-guided interventions are the studies of Veldeman et al. They evaluated outcome between periods before and after introducing an invasive MMM-guided protocol to avoid PbtO_2_ < 10 mmHg and CMD L/P-ratio > 40 in severe SAH patients with suspicion of delayed cerebral ischemia (Veldeman et al., 2020a; Veldeman et al., 2020b).

**FIGURE 4:**
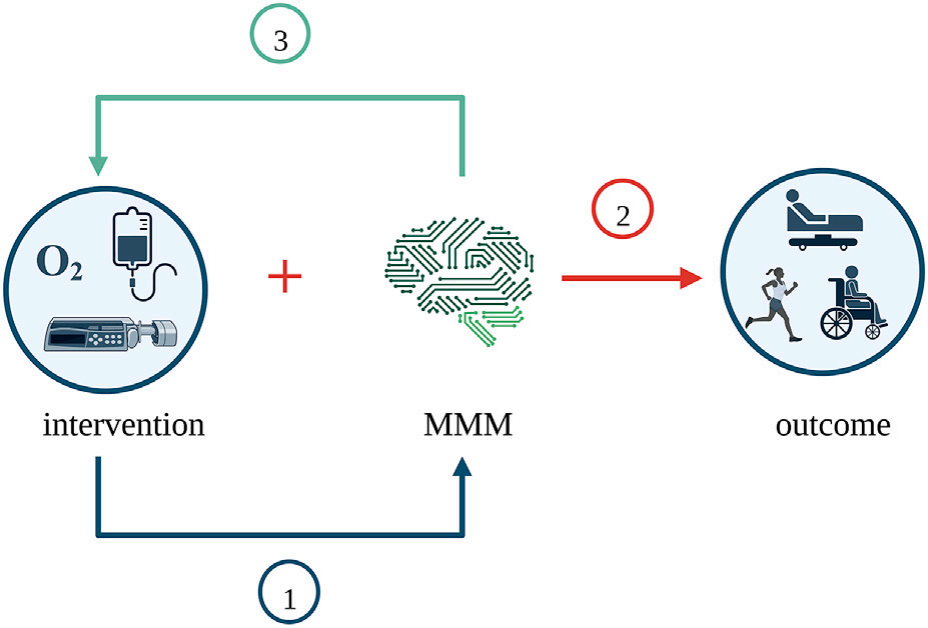
Purposes of interventions across the MMM studies. MMM was examined in three different ways across the studies. Firstly, MMM was the outcome, and the intervention’s effectiveness was studied. Secondly, MMM was considered along with the intervention for its effect on clinical outcome. Thirdly, thresholds of MMM were used to dictate intervention, and the need for intervention was studied. Created with BioRender.com. MMM, multimodality monitoring.

Interventions in the MMM studies serve mainly three purposes. Firstly, monitoring the effectiveness of an intervention. Secondly, collecting monitoring data in combination with an intervention for outcome evaluation/prediction. A third purpose is monitoring the need for an intervention. In other words, interventions guided by MMM to investigate the interplay between monitoring and a combination of (in general, systemic) interventions. Figure 4 illustrates the purposes of the interventions across the MMM studies.

To give insight into the range of systemic-, cerebral-, and MMM-guided interventions, we classified them into nine categories: ABP management, biomarkers, fluid management, mixed (combination of different) interventions, RBC-transfusion, physical (movement) interventions, vasospasm therapy, ventilation management, and other interventions. The number of studies per group is mostly less than five. The largest groups are the mixed interventions used to guide MMM (11 studies), followed by ventilation management interventions (10 studies). On the other hand, biomarkers and physical (movement) interventions were studied in only three studies. The specific interventions and the corresponding number of studies per category are shown in Table 4.

**TABLE 4:** Systemic-, cerebral- interventions, and interventions guided by cerebral multimodality monitoring in 44 studies.

Intervention group	Interventions	No. of studies	References
ABP-management	Arterial blood pressure-management	4	(Jakkula et al., 2018; Calviello et al., 2019; Sekhon et al., 2019; Kovacs et al., 2021)^b^
Biomarkers	Neuroglobin, prostacyclin, succinate	3	(Ding et al., 2019; Koskinen et al., 2019; Khellaf et al., 2022)^a^
Fluid management	CSF drainage, hypertonic saline, and/or fluid management	5	(Akbik et al., 2017; Carteron et al., 2018; Hoiland et al., 2021; Rass et al., 2021; Bernini et al., 2022)
Mixed interventions	Nimodipine, ICP/CPP management (vasopressors, sedation, etc.), hyperoxia, glucose, head elevation/flat position	11	(Bele et al., 2015; Lin et al., 2015; Okonkwo et al., 2017; Sekhon et al., 2017; Rass et al., 2019; Veldeman et al., 2020a; Veldeman et al., 2020b; Fergusson et al., 2021; Gouvea Bogossian et al., 2021; Khellaf et al., 2022; Winberg et al., 2022)^a^
RBC transfusion	Red blood cell transfusion	4	(Sekhon et al., 2015; Kurtz et al., 2016; McCredie et al., 2017; Gouvêa Bogossian et al., 2022)
Physical (movement) interventions	Intrahospital transport, head elevation/flat position	3	(Burnol et al., 2021; Dagod et al., 2021; Hosmann et al., 2021)
Vasospasm therapy	Nimodipine, endovascular therapy, papaverine-hydrochloride	4	(Hockel et al., 2016; Albanna et al., 2017; Hockel et al., 2017; Hosmann et al., 2020)
Ventilation management	Hyperoxia, hypercapnia, hypocapnia	10	(Westermaier et al., 2016; Zhang et al., 2016; Ghosh et al., 2017; Sahoo et al., 2017; Brandi et al., 2019; Calviello et al., 2019; Svedung Wettervik et al., 2020b; Stetter et al., 2021; Gargadennec et al., 2022; Hosmann et al., 2022)^b^
Other therapies	Analgesia, hypothermia, enteral nutrition, decompressive craniectomy	4	(Flynn et al., 2015; Kofler et al., 2018; Kovac et al., 2018; Ianosi et al., 2020)

^a^ One study included both cerebral intervention and MMM-guided treatment (mixed interventions).

^b^ One study included two study groups studying two interventions.

ABP, arterial blood pressure; CSF, cerebral spinal fluid; RBC, red blood cell; No., number; ICP, intracranial pressure; CPP, cerebral perfusion pressure; MMM, multimodality monitoring.

## 8 Results objective III: Clinical outcome in interventional studies

Clinical outcome is a study endpoint in 18 (41%) of the 44 interventional studies. Systemic- and cerebral interventions evaluated MMM for either outcome prediction (1 study) (Lubillo et al., 2018) or monitoring the effectiveness of an intervention in both the MMM signals and clinical outcome (8 studies) (Hockel et al., 2016; Jakkula et al., 2018; Ding et al., 2019; Sekhon et al., 2019; Svedung Wettervik et al., 2020b; Dagod et al., 2021; Kovacs et al., 2021). For MMM-guided intervention studies, clinical outcome resulted from the interplay between MMM and interventions. Nine (9/11, 82%) of the MMM-guided included clinical outcome as an endpoint, of which seven showed an improved outcome in favor of the MMM-guided group (78%, 7/9 studies). Five (45%) studied an ICP and PbtO_2_-guided treatment in either TBI (Lin et al., 2015; Okonkwo et al., 2017; Sekhon et al., 2017) or SAH (Rass et al., 2019; Gouvea Bogossian et al., 2021) patients. Two of these compared pre-/post implementation of an MMM-guided protocol. Okonkwo et al. (2017) studied the feasibility and safety of an ICP and PbtO_2_ protocol in a randomized controlled trial (RCT). Their study showed lower mortality and improved outcome, but the effects did not reach statistical significance. This was attributed to the small sample size. In addition, Rass et al. (2019) studied the brain hypoxia burden in two centers and found no difference in PbtO_2_-levels and clinical outcome. The remaining four MMM-guided studies that showed an improved clinical outcome included the following modalities: (I) CMD in combination with ICP, PbtO_2,_ TCD (Veldeman et al., 2020a; Veldeman et al., 2020b) (II) ICP, PbtO_2_, Cerebral T, and SvjO_2_ (Fergusson et al., 2021), and (III) ICP, PbtO_2,_ rCBF, and TCD (Bele et al., 2015).

## 9 Discussion

The principal insights gained from our analysis of the MMM literature are that: (Insight I) most reports of MMM involve just two monitoring modalities, one of which is typically ICP monitoring; (Insight II) we found relatively often 10 (8.9%) ECoG and 7 (6.3%) dEEG studies, of which 8 (50%) investigated cortical spreading depolarization; (Insight III) our results show that MMM is primarily used in TBI and SAH patients. In addition, ICH and AIS are sparsely studied as a single study population but mainly combined with other acute brain injuries. One of the reasons could be that (non) invasive cerebral monitoring was not part of HIBI, AIS, and ICH (international) treatment guidelines and protocols compared to TBI and SAH patients; (Insight IV) most MMM studies had an observational design without direct clinical and therapeutic implications at the bedside; (Insight V) The sample sizes are in general small with long inclusion periods; (Insight VI) a large variety of interventions were studied in limited numbers of studies; (Insight VII) seven of the nine MMM-guided intervention studies showed a significant improved clinical outcome in favor of treatment guided by MMM.

### 9.1 Strengths and weaknesses of MMM (studies)

#### 9.1.1 Acceptance of MMM in clinical practice

Almost 10% of the studies were MMM-guided, of which only one was an RCT. The remaining MMM-guided studies investigated a clinical intervention protocol guided by MMM (e.g., comparing the pre-/post implementation of a protocol). This reflects the acceptance of MMM in current clinical practice, even with general lack of Class I evidence. The recent Seattle International Severe Traumatic Brain Injury Consensus Conference (SIBICC) included in their tier-based protocol not only ICP but also PbtO_2_ for monitoring (Hawryluk et al., 2019). While no (phase-III) clinical outcome benefits of MMM-guided treatment exist yet, there are three large phase-III trials currently underway. All study in TBI patients whether a combined ICP and PbtO_2_-guided tiered management protocol is associated with a beneficial outcome (ClinicalTrials.gov, 2021a; ClinicalTrials.gov, 2021b; Udy, 2021). ICP and PbtO_2_ monitoring were also mostly applied in the MMM studies. This is not surprising as ICP/CPP monitoring is the cornerstone of TBI monitoring and treatment guidelines (Carney et al., 2017; Hawryluk et al., 2019). sEEG has infrequently been used, which is surprising as non-convulsive status epilepticus has been reported in 10–20% of NICU patients (Laccheo et al., 2015). Epileptic activity is not only related to cortical damage and poor outcome but might also confound the interpretation of MMM results (Nolan et al., 2021). The least studied modality is SvjO_2._ Although SvjO_2_ has a lengthy history of use, the availability of non-invasive alternatives like NIRS or the increasing use of PbtO_2_ may explain this (Bhatia and Gupta, 2007).

#### 9.1.2 Multiple research questions per study cohort

Our results showed that limited (20%) multicenter studies were included, of which more than ten concerned COSBID (Co-Operative studies on Brain Injury Depolarizations) or CENTER-TBI (Collaborative European Neuro Trauma Effectiveness Research in TBI) study cohorts. Both cohorts are collaborations between different international centers studying a diversity of research questions. In addition, single-center studies also reuse their cohort by publishing different research questions, for example, the series from Svedung Wettervik et al. (Svedung Wettervik et al., 2019; Svedung Wettervik et al., 2020b; Svedung Wettervik et al., 2020a). The strength of a recycled study cohort is that it saves time and money; and could result in a broad understanding of the neuromonitoring signals. Also, the different studies were performed under the same conditions, which improves the ability to compare the studies. On the other hand, the weakness is that reusing study cohorts overestimate the feasibility of MMM for clinical use.

#### 9.1.3 Data quality

We found that 30% of the studies enrolled patients over a period of more than 5 years. The long inclusion period, in combination with the low number of patients, might be explained because several studies use large (observational) databases to select patients with a particular condition (e.g., ICP plateau waves). In addition, insufficient data quality might contribute. A number of studies excluded patients due to poor data quality of both invasive and non-invasive monitoring modalities. For example, rCBF monitoring (Hemedex Inc.; Cambridge, MA) requires regular calibrations, which causes a regular artifact in the data, whereby Foreman et al. could use only 62% of the rCBF monitoring time (Foreman et al., 2018). Other examples are the exclusion of five (21%) NIRS data recordings (McCredie et al., 2017); the exclusion of five (4.8%) PbtO_2_ recordings due to malfunctioning PbtO_2_ probes (Rass et al., 2019); the exclusion of 17 (10%) recordings because of poor ECoG data quality (Hartings et al., 2020); and exclusion of 8.8% (637/7223) of the hourly analyzed CMD samples because of insufficient quality (Winberg et al., 2022). Finally, 30% (100–2435/3483 h) of the ICP and Cerebral T data was excluded due to artifacts (Birg et al., 2021). Misplaced probes were less often reported (Gagnon et al., 2020; Winberg et al., 2022) but also contributed to the removal of patient data. A weakness of MMM (studies) is that although most studies were performed in NICU, collecting continuous, high-quality data from multiple monitors seems complex as several studies report artifacts or poor data quality, limiting its feasibility in clinical practice. Moreover, post-hoc manual removal of a large number of artifacts lead to a false clinical conclusion.

#### 9.1.4 Data duration and the start of monitoring

The data covered for analysis for more than 24 h of monitoring was only 58%. The short analysis periods contrast with continuous or regularly daily updated monitoring data. Important to realize is that we used the data analysis period for comparisons instead of the total monitoring period (of which data were limited reported). The short analysis periods are related to, firstly, the type of monitoring. For example, 79% (15/19 studies) of the TCD studies reported time periods <24 h of monitoring. Recent technological advances in automated stable TCD insonations will probably allow longer recordings (Zeiler et al., 2019). Secondly, the study design. For example, studies selected monitoring epochs around specific interventions or physiological changes (such as pre-/post-hypocapnia intervention) (Brandi et al., 2019) or pathophysiological insults (such as delayed cerebral ischemia) (Patet et al., 2017). Thirdly, the timing of the applied monitoring (if reported) after the estimated time of ictus. The strength of MMM would be to have continuous monitoring available, informing about different aspects of the brain and evaluate changes over time. However, since limited studies analyze whole signal recordings and very few studies reported the delay between the estimated time of ictus and the start of study monitoring, it is a weakness of the current MMM studies that it is often unknown which pathophysiological condition the patients were studied in time. Therefore, we recommend to report the disease time course for multimodality studies. In this way, we will gain insight into time-specific monitoring patterns related to pathophysiological changes.

#### 9.1.5 Signal integration

We defined MMM as “the application and reporting results of at least two modalities (i.e., modalities were part of the research protocol) without aiming for superiority/inferiority between modalities”. However, almost 30% of the studies monitored patients with additional neuromonitoring modalities for other (clinical) purposes. Therefore, the results included these additional modalities as “other modalities”. For example, ICP monitoring is standard of care in TBI patients and has been reported only in the methods of the study as part of their “clinical management”. However, when the aim or objective(s) of the study was to study the relationship between CMD and PbtO_2_, ICP was not classified as part of their study modalities.

The strength of MMM would be to integrate multiple monitoring signals. However, we observed that the analysis was mainly group comparisons, correlations, and uni- or multivariate (regression) analysis. Hemphill et al. proposed advanced analysis in NICU in 2011. They discussed that advanced analysis can be divided into unsupervised data-driven (e.g., hierarchical clustering), supervised data-driven (e.g., decision trees, neural networks), or model-based methods (e.g., dynamic system models Dynamic Bayesian networks). Regression analysis is also part of data-driven methods, but these are only appropriate for linear predictions (Hemphill et al., 2011; Volovici et al., 2022), whereas time series of different modalities include multiple features (dimensions) and interactions. For these complex interactions, model-based methods are more appropriate (Hemphill et al., 2011; Acosta et al., 2022). We included an explorative study using hierarchical clustering (Rajagopalan et al., 2022). They successfully classified four clusters, each corresponding with a specific (patho)-physiological state (cerebral ischemia, intracranial hypertension without ischemia, hyper-glycolysis, and normal cerebral physiology) from cerebral MMM data. In addition, Åkerlund et al. (2022) applied an unsupervised statistical clustering model on clinical variables in a TBI population. They concluded that this approach might contribute to a refinement in disease classification and a better understanding of pathological processes and their relation with clinical outcome. For future studies, it might be interesting to integrate different domains such as neuromonitoring data, clinical variables, medication (e.g., sedatives, analgesia, vasopressor medication), ventilation, or advanced cardiac monitoring signals for a further understanding of complex disease entities. However, for successful models, a large number of patients with complete and annotated data sets are required (Acosta et al., 2022).

### 9.2 Limitations

Our current MMM overview is based on a stepwise search covering a 7 years period. However, we should acknowledge that this approach has limitations. Firstly, we studied the literature starting from the projections of Le Roux et al. to give an overview of the literature, knowing it limits conclusions about MMM advances over time. In addition, only adult patients were included, while reviewing pediatric studies would be of interest too. Secondly, although the review outline and interpretation of the review results were discussed within the coauthors’ group, the studies were screened and classified by a single author. In addition, we did not use a formal (PRISMA-guided) systematic review and meta-analysis, given the heterogeneity in study design, patient population, and monitoring devices applied. However, we performed a reproducible and extensive literature search with pre-defined inclusion criteria covering the past 7 years.

### 9.3 Future perspectives

For the upcoming years, it would be recommended to focus on, firstly, data quality, collection of both MMM signals and other continuous trends (medication, ventilation, advanced cardiac monitoring), and advanced analytics. Interdisciplinary collaborations can achieve this. Secondly, increasing sample sizes, homogeneity of studied diseases, and shortening inclusion periods. This can be achieved by increasing the number of multicenter studies. Thirdly, introducing new refined definitions of secondary injuries to improve the comparison between studies. Fourthly, one of the stated near future MMM reflections was the increased validation of direct current EEG methodology (i.e., the ability to detect a wide range of EEG frequencies) (Kovac et al., 2018) to detect cortical spreading depolarization. The included explorative studies showed promising results regarding the pathophysiology of cortical spreading depolarization. Therefore, future exploration could indicate a potential new treatment target for acute brain injury patients (Winkler et al., 2017; Hartings et al., 2020); and, finally, the start of new phase-III MMM studies that might result in new outcome benefits and therapies for acute brain injured patients*.*

## 10 Conclusion

Cerebral MMM in neurocritical care patients with acute brain injury focuses predominantly on bimodal monitoring, studied mainly in TBI and SAH patients. Definitions of secondary injuries are limited by the number of modalities and differ in entity due to different thresholds. In addition, the applied interventions are large in variety, but they are limited in the number of studies. Although the improved clinical outcome in MMM-guided intervention studies strengthens the belief in this application, further interdisciplinary collaborations are needed to overcome the heterogeneity. Future research should focus on improved data collection, sample sizes, refining definitions of secondary injuries, and standardized interventions. Only then can we proceed with complex outcome studies with MMM-guided treatment.

## References

[B1] Acosta J. N. Falcone G. J. Rajpurkar P. Topol E. J. (2022). Multimodal biomedical AI. Nat. Med. 28 (9), 1773–1784. 10.1038/s41591-022-01981-2 36109635

[B2] Akbik O. S. Krasberg M. Nemoto E. M. Yonas H. (2017). Effect of cerebrospinal fluid drainage on brain tissue oxygenation in traumatic brain injury. J. Neurotrauma 34 (22), 3153–3157. 10.1089/neu.2016.4912 28614970

[B3] Åkerlund C. A. I. Holst A. Stocchetti N. Steyerberg E. W. Menon D. K. Ercole A. (2022). Clustering identifies endotypes of traumatic brain injury in an intensive care cohort: A CENTER-TBI study. Crit. Care 26 (1), 228. 10.1186/s13054-022-04079-w 35897070 PMC9327174

[B4] Al-Mufti F. Lander M. Smith B. Morris N. A. Nuoman R. Gupta R. (2019). Multimodality monitoring in neurocritical care: Decision-making utilizing direct and indirect surrogate markers. J. Intensive Care Med. 34 (6), 449–463. 10.1177/0885066618788022 30205730

[B5] Albanna W. Weiss M. Müller M. Brockmann M. A. Rieg A. Conzen C. (2017). Endovascular rescue therapies for refractory vasospasm after subarachnoid hemorrhage: A prospective evaluation study using multimodal, continuous event neuromonitoring. Neurosurgery 80 (6), 942–949. 10.1093/neuros/nyw132 28201539

[B6] Bele S. Proescholdt M. A. Hochreiter A. Schuierer G. Scheitzach J. Wendl C. (2015). Continuous intra-arterial nimodipine infusion in patients with severe refractory cerebral vasospasm after aneurysmal subarachnoid hemorrhage: A feasibility study and outcome results. Acta Neurochir. 157 (12), 2041–2050. 10.1007/s00701-015-2597-z 26439105

[B7] Bernini A. Miroz J-P. Abed-Maillard S. Favre E. Iaquaniello C. Ben-Hamouda N. (2022). Hypertonic lactate for the treatment of intracranial hypertension in patients with acute brain injury. Sci. Rep. 12 (1), 3035. 10.1038/s41598-022-07129-z 35194150 PMC8864009

[B8] Bhatia A. Gupta A. K. (2007). Neuromonitoring in the intensive care unit. II. Cerebral oxygenation monitoring and microdialysis. Intensive Care Med. 33 (8), 1322–1328. 10.1007/s00134-007-0660-9 17522846

[B9] Birg T. Ortolano F. Wiegers E. J. A. Smielewski P. Savchenko Y. Ianosi B. A. (2021). Brain temperature influences intracranial pressure and cerebral perfusion pressure after traumatic brain injury: A CENTER-TBI study. Neurocrit. Care 35 (3), 651–661. 10.1007/s12028-021-01294-1 34331210 PMC8692292

[B10] Brandi G. Stocchetti N. Pagnamenta A. Stretti F. Steiger P. Klinzing S. (2019). Cerebral metabolism is not affected by moderate hyperventilation in patients with traumatic brain injury. Crit. Care 23 (1), 45. 10.1186/s13054-018-2304-6 30760295 PMC6375161

[B11] Burnol L. Payen J-F. Francony G. Skaare K. Manet R. Morel J. (2021). Impact of head-of-bed posture on brain oxygenation in patients with acute brain injury: A prospective cohort study. Neurocrit. Care 35 (3), 662–668. 10.1007/s12028-021-01240-1 34312789 PMC8312355

[B12] Calviello L. A. Zeiler F. A. Donnelly J. Uryga A. de Riva N. Smielewski P. (2019). Estimation of pulsatile cerebral arterial blood volume based on transcranial Doppler signals. Med. Eng. Phys. 74, 23–32. 10.1016/j.medengphy.2019.07.019 31648880

[B13] Carney N. Totten A. M. O’Reilly C. Ullman J. S. Hawryluk G. W. J. Bell M. J. (2017). Guidelines for the management of severe traumatic brain injury, fourth edition. Neurosurgery 80 (1), 6–15. 10.1227/NEU.0000000000001432 Fourth Edition 27654000

[B14] Carteron L. Solari D. Patet C. Quintard H. Miroz J-P. Bloch J. (2018). Hypertonic lactate to improve cerebral perfusion and glucose availability after acute brain injury. Crit. Care Med. 46 (10), 1649–1655. 10.1097/CCM.0000000000003274 29923931

[B15] ClinicalTrials.gov (2021). Brain oxygen optimization in severe TBI (BOOST3): A comparative effectiveness study to test the efficacy of a prescribed treatment protocol based on monitoring the partial pressure of brain tissue tissue oxygen. NCT03754114 [Internet]. [cited 2021 1]. Available from: https://clinicaltrials.gov/ct2/show/NCT03754114.

[B16] ClinicalTrials.gov (2021). Impact of early optimization of brain oxygenation on neurological outcome after severe traumatic brain injury (OXY-TC). NCT02754063 [Internet]. [cited 2021 1]. Available from: https://clinicaltrials.gov/ct2/show/NCT02754063.

[B17] Cnossen M. C. Huijben J. A. van der Jagt M. Volovici V. van Essen T. Polinder S. (2017). Variation in monitoring and treatment policies for intracranial hypertension in traumatic brain injury: A survey in 66 neurotrauma centers participating in the CENTER-TBI study. Crit. Care 21 (1), 233. 10.1186/s13054-017-1816-9 28874206 PMC5586023

[B18] Dagod G. Roustan J. P. Bringuier-Branchereau S. Ridolfo J. tinez O. Capdevila X. (2021). Effect of a temporary lying position on cerebral hemodynamic and cerebral oxygenation parameters in patients with severe brain trauma. Acta Neurochir. (Wien). 163 (9), 2595–2602. 10.1007/s00701-021-04851-x 34236525

[B19] Ding C. Y. Kang D. Z. Wang Z. L. Lin Y. X. Jiang C. Z. Yu L. H. (2019). Serum ngb (neuroglobin) is associated with brain metabolism and functional outcome of aneurysmal subarachnoid hemorrhage. Stroke 50 (7), 1887–1890. 10.1161/STROKEAHA.119.025733 31182001

[B20] Fergusson N. A. Hoiland R. L. Thiara S. Foster D. Gooderham P. Rikhraj K. (2021). Goal-directed care using invasive neuromonitoring versus standard of care after cardiac arrest: A matched cohort study. Crit. Care Med. 49 (8), 1333–1346. 10.1097/CCM.0000000000004945 33711002

[B21] Flynn L. M. C. Rhodes J. Andrews P. J. D. (2015). Therapeutic hypothermia reduces intracranial pressure and partial brain oxygen tension in patients with severe traumatic brain injury: Preliminary data from the Eurotherm3235 trial. Ther. Hypothermia Temp. Manag. 5 (3), 143–151. 10.1089/ther.2015.0002 26060880 PMC4575517

[B22] Foreman B. Ngwenya L. B. Stoddard E. Hinzman J. M. Andaluz N. Hartings J. A. (2018). Safety and reliability of bedside, single burr hole technique for intracranial multimodality monitoring in severe traumatic brain injury. Neurocrit. Care 29 (3), 469–480. 10.1007/s12028-018-0551-7 29949001

[B23] Gagnon A. Laroche M. Williamson D. Giroux M. Giguère J-F. Bernard F. (2020). Incidence and characteristics of cerebral hypoxia after craniectomy in brain-injured patients: A cohort study. J. Neurosurg., 1–8. 10.3171/2020.6.JNS20776 33157533

[B24] Gargadennec T. Ferraro G. Chapusette R. Chapalain X. Bogossian E. Van Wettere M. (2022). Detection of cerebral hypoperfusion with a dynamic hyperoxia test using brain oxygenation pressure monitoring. Crit. Care 26 (1), 35. 10.1186/s13054-022-03918-0 35130953 PMC8822803

[B25] Ghosh A. Highton D. Kolyva C. Tachtsidis I. Elwell C. E. Smith M. (2017). Hyperoxia results in increased aerobic metabolism following acute brain injury. J. Cereb. Blood Flow. Metab. 37 (8), 2910–2920. 10.1177/0271678X16679171 27837190 PMC5536254

[B26] Gouvea Bogossian E. Diaferia D. Ndieugnou Djangang N. Menozzi M. Vincent J-L. Talamonti M. (2021). Brain tissue oxygenation guided therapy and outcome in non-traumatic subarachnoid hemorrhage. Sci. Rep. 11 (1), 16235. 10.1038/s41598-021-95602-6 34376735 PMC8355344

[B27] Gouvêa Bogossian E. Rass V. Lindner A. Iaquaniello C. Miroz J. P. Cavalcante Dos Santos E. (2022). Factors associated with brain tissue oxygenation changes after RBC transfusion in acute brain injury patients. Crit. Care Med. 50 (6), e539–e547. 10.1097/CCM.0000000000005460 35132018

[B28] Hartings J. A. Andaluz N. Bullock M. R. Hinzman J. M. Mathern B. Pahl C. (2020). Prognostic value of spreading depolarizations in patients with severe traumatic brain injury. JAMA Neurol. 77 (4), 489–499. 10.1001/jamaneurol.2019.4476 31886870 PMC6990808

[B29] Hawryluk G. W. J. Aguilera S. Buki A. Bulger E. Citerio G. Cooper D. J. (2019). A management algorithm for patients with intracranial pressure monitoring: The Seattle international severe traumatic brain injury Consensus conference (SIBICC). Intensive Care Med. 45 (12), 1783–1794. 10.1007/s00134-019-05805-9 31659383 PMC6863785

[B30] Hemphill J. C. Andrews P. De Georgia M. (2011). Multimodal monitoring and neurocritical care bioinformatics. Nat. Rev. Neurol. 7 (8), 451–460. 10.1038/nrneurol.2011.101 21750522

[B31] Hockel K. Diedler J. Steiner J. Birkenhauer U. Danz S. Ernemann U. (2016). Long-term, continuous intra-arterial nimodipine treatment of severe vasospasm after aneurysmal subarachnoid hemorrhage. World Neurosurg. 88, 104–112. 10.1016/j.wneu.2015.11.081 26732964

[B32] Hockel K. Diedler J. Steiner J. Birkenhauer U. Ernemann U. Schuhmann M. U. (2017). Effect of intra-arterial and intravenous nimodipine therapy of cerebral vasospasm after subarachnoid hemorrhage on cerebrovascular reactivity and oxygenation. World Neurosurg. 101, 372–378. 10.1016/j.wneu.2017.02.014 28232152

[B33] Hoiland R. L. Ainslie P. N. Wellington C. L. Cooper J. Stukas S. Thiara S. (2021). Brain hypoxia is associated with neuroglial injury in humans post-cardiac arrest. Circ. Res. 129 (5), 583–597. 10.1161/CIRCRESAHA.121.319157 34287000 PMC8376277

[B34] Hosmann A. Angelmayr C. Hopf A. Rauscher S. Brugger J. Ritscher L. (2021). Detrimental effects of intrahospital transport on cerebral metabolism in patients suffering severe aneurysmal subarachnoid hemorrhage. J. Neurosurg. 1–8, 1–8. 10.3171/2020.8.JNS202280 33711812

[B35] Hosmann A. Schnackenburg P. Rauscher S. Hopf A. Bohl I. Engel A. (2022). Brain tissue oxygen response as indicator for cerebral lactate levels in aneurysmal subarachnoid hemorrhage patients. J. Neurosurg. Anesthesiol. 34 (2), 193–200. 10.1097/ANA.0000000000000713 32701532

[B36] Hosmann A. Wang W-T. Dodier P. Bavinzski G. Engel A. Herta J. (2020). The impact of intra-arterial papaverine-hydrochloride on cerebral metabolism and oxygenation for treatment of delayed-onset post-subarachnoid hemorrhage vasospasm. Neurosurgery 87 (4), 712–719. 10.1093/neuros/nyz500 31792510

[B37] Hutchinson P. J. Jalloh I. Helmy A. Carpenter K. L. H. Rostami E. Bellander B. M. (2015). Consensus statement from the 2014 international microdialysis forum. Intensive Care Med. 41 (9), 1517–1528. 10.1007/s00134-015-3930-y 26194024 PMC4550654

[B38] Ianosi B. Rass V. Gaasch M. Huber L. Lindner A. Hackl W. O. (2020). An observational study on the use of intravenous non-opioid analgesics and antipyretics in poor-grade subarachnoid hemorrhage: Effects on hemodynamics and systemic and brain temperature. Ther. Hypothermia Temp. Manag. 10 (1), 27–36. 10.1089/ther.2018.0046 30835164

[B39] Jakkula P. Pettilä V. Skrifvars M. B. Hästbacka J. Loisa P. Tiainen M. (2018). Targeting low-normal or high-normal mean arterial pressure after cardiac arrest and resuscitation: A randomised pilot trial. Intensive Care Med. 44, 2091–2101. 10.1007/s00134-018-5446-8 30443729 PMC6280836

[B40] Khellaf A. Garcia N. M. Tajsic T. Alam A. Stovell M. G. Killen M. J. (2022). Focally administered succinate improves cerebral metabolism in traumatic brain injury patients with mitochondrial dysfunction. J. Cereb. Blood Flow. Metab. 42 (1), 39–55. 10.1177/0271678X211042112 34494481 PMC8721534

[B41] Kofler M. Schiefecker A. J. Beer R. Gaasch M. Rhomberg P. Stover J. (2018). Enteral nutrition increases interstitial brain glucose levels in poor-grade subarachnoid hemorrhage patients. J. Cereb. Blood Flow. Metab. 38 (3), 518–527. 10.1177/0271678X17700434 28322077 PMC5851142

[B42] Koskinen L-O. D. Sundström N. Hägglund L. Eklund A. Olivecrona M. (2019). Prostacyclin affects the relation between brain interstitial glycerol and cerebrovascular pressure reactivity in severe traumatic brain injury. Neurocrit. Care 31 (3), 494–500. 10.1007/s12028-019-00741-4 31123992 PMC6872514

[B43] Kovac S. Speckmann E-J. Gorji A. (2018). Uncensored EEG: The role of DC potentials in neurobiology of the brain. Prog. Neurobiol. 165–167, 51–65. 10.1016/j.pneurobio.2018.02.001 29428834

[B44] Kovacs M. Peluso L. Njimi H. De Witte O. Gouvêa Bogossian E. Quispe Cornejo A. (2021). Optimal cerebral perfusion pressure guided by brain oxygen pressure measurement. Front. Neurol. 12, 732830. 10.3389/fneur.2021.732830 34777201 PMC8581172

[B45] Krzywinski M. I. Schein J. E. Birol I. Connors J. Gascoyne R. Horsman D. (2009). Circos: An information aesthetic for comparative genomics. Genome Res. 19 (9), 1639–1645. 10.1101/gr.092759.109 19541911 PMC2752132

[B46] Kurtz P. Helbok R. Claassen J. Schmidt J. M. Fernandez L. Stuart R. M. (2016). The effect of packed red blood cell transfusion on cerebral oxygenation and metabolism after subarachnoid hemorrhage. Neurocrit. Care 24 (1), 118–121. 10.1007/s12028-015-0180-3 26195087

[B47] Laccheo I. Sonmezturk H. Bhatt A. B. Tomycz L. Shi Y. Ringel M. (2015). Non-convulsive status epilepticus and non-convulsive seizures in neurological ICU patients. Neurocrit. Care 22 (2), 202–211. 10.1007/s12028-014-0070-0 25246236

[B48] Le Roux P. Menon D. K. Citerio G. Vespa P. Bader M. K. Brophy G. M. (2014). Consensus summary statement of the international multidisciplinary Consensus conference on multimodality monitoring in neurocritical care: A statement for healthcare professionals from the neurocritical care society and the European society of intensive care medicine. Neurocrit. Care 21 (2), 1–26. 10.1007/s12028-014-0041-5 PMC10596301 25208678

[B49] Lin C-M. Lin M-C. Huang S-J. Chang C-K. Chao D-P. Lui T-N. (2015). A prospective randomized study of brain tissue oxygen pressure-guided management in moderate and severe traumatic brain injury patients. Biomed. Res. Int. 2015, 529580. 10.1155/2015/529580 26413530 PMC4564619

[B83] Lindner A. Rass V. Ianosi B-A. Schiefecker A. J. Kofler M. Gaasch M. (2021). Individualized blood pressure targets in the postoperative care of patients with intracerebral hemorrhage. J. Neurosurg., 1–10. 10.3171/2020.9.JNS201024 33836501

[B50] Lubillo S. T. Parrilla D. M. Blanco J. Morera J. Dominguez J. Belmonte F. (2018). Prognostic value of changes in brain tissue oxygen pressure before and after decompressive craniectomy following severe traumatic brain injury. J. Neurosurg. 128 (5), 1538–1546. 10.3171/2017.1.JNS161840 28665250

[B51] Makarenko S. Griesdale D. E. Gooderham P. Sekhon M. S. (2016). Multimodal neuromonitoring for traumatic brain injury: A shift towards individualized therapy. J. Clin. Neurosci. 26, 8–13. 10.1016/j.jocn.2015.05.065 26755455

[B52] McCredie V. A. Piva S. Santos M. Xiong W. de Oliveira Manoel A. L. Rigamonti A. (2017). The impact of red blood cell transfusion on cerebral tissue oxygen saturation in severe traumatic brain injury. Neurocrit. Care 26 (2), 247–255. 10.1007/s12028-016-0310-6 27757915

[B53] Nolan J. P. Sandroni C. Böttiger B. W. Cariou A. Cronberg T. Friberg H. (2021). European resuscitation council and European society of intensive care medicine guidelines 2021: Post-resuscitation care. Resuscitation 161, 220–269. 10.1016/j.resuscitation.2021.02.012 33773827

[B54] Nyholm L. Howells T. Lewén A. Hillered L. Enblad P. (2017). The influence of hyperthermia on intracranial pressure, cerebral oximetry, and cerebral metabolism in traumatic brain injury. Ups. J. Med. Sci. 122 (3), 177–184. 10.1080/03009734.2017.1319440 28463046 PMC5649323

[B55] Okonkwo D. O. Shutter L. A. Moore C. Temkin N. R. Puccio A. M. Madden C. J. (2017). Brain oxygen optimization in severe traumatic brain injury phase-II: A phase II randomized trial*. Crit. Care Med. 45 (11), 1907–1914. 10.1097/CCM.0000000000002619 29028696 PMC5679063

[B56] Patet C. Quintard H. Zerlauth J-B. Maibach T. Carteron L. Suys T. (2017). Bedside cerebral microdialysis monitoring of delayed cerebral hypoperfusion in comatose patients with poor grade aneurysmal subarachnoid haemorrhage. J. Neurol. Neurosurg. Psychiatry 88 (4), 332–338. 10.1136/jnnp-2016-313766 27927702

[B57] Rajagopalan S. Baker W. Mahanna-Gabrielli E. Kofke A. W. Balu R. (2022). Hierarchical cluster Analysis identifies distinct physiological states after acute brain injury. Neurocrit. Care 36 (2), 630–639. 10.1007/s12028-021-01362-6 34661861 PMC11346511

[B58] Rass V. Bogossian E. G. Ianosi B-A. Peluso L. Kofler M. Lindner A. (2021). The effect of the volemic and cardiac status on brain oxygenation in patients with subarachnoid hemorrhage: A bi-center cohort study. Ann. Intensive Care 11 (1), 176. 10.1186/s13613-021-00960-z 34914011 PMC8677880

[B59] Rass V. Solari D. Ianosi B. Gaasch M. Kofler M. Schiefecker A. J. (2019). Protocolized brain oxygen optimization in subarachnoid hemorrhage. Neurocrit. Care 31 (2), 263–272. 10.1007/s12028-019-00753-0 31218640 PMC6757026

[B60] Sahoo S. Sheshadri V. Sriganesh K. Madhsudana Reddy K. R. Radhakrishnan M. Umamaheswara Rao G. S. (2017). Effect of hyperoxia on cerebral blood flow velocity and regional oxygen saturation in patients operated on for severe traumatic brain injury-the influence of cerebral blood flow autoregulation. World Neurosurg. 98, 211–216. 10.1016/j.wneu.2016.10.116 27810458

[B61] Sekhon M. S. Ainslie P. N. Menon D. K. Thiara S. S. Cardim D. Gupta A. K. (2020). Brain hypoxia secondary to diffusion limitation in hypoxic ischemic brain injury postcardiac arrest. Crit. Care Med. 48 (3), 378–384. 10.1097/CCM.0000000000004138 31789834

[B62] Sekhon M. S. Gooderham P. Menon D. K. Brasher P. M. A. Foster D. Cardim D. (2019). The burden of brain hypoxia and optimal mean arterial pressure in patients with hypoxic ischemic brain injury after cardiac arrest. Crit. Care Med. 47 (7), 960–969. 10.1097/CCM.0000000000003745 30889022

[B63] Sekhon M. S. Gooderham P. Toyota B. Kherzi N. Hu V. Dhingra V. K. (2017). Implementation of neurocritical care is associated with improved outcomes in traumatic brain injury. Can. J. Neurol. Sci. 44 (4), 350–357. 10.1017/cjn.2017.25 28343456

[B64] Sekhon M. S. Griesdale D. E. Czosnyka M. Donnelly J. Liu X. Aries M. J. (2015). The effect of red blood cell transfusion on cerebral autoregulation in patients with severe traumatic brain injury. Neurocrit. Care 23 (2), 210–216. 10.1007/s12028-015-0141-x 25894454

[B65] Smith M. (2018). Multimodality neuromonitoring in adult traumatic brain injury: A narrative review. Anesthesiology 128 (2), 401–415. 10.1097/ALN.0000000000001885 28938277

[B66] Stetter C. Weidner F. Lilla N. Weiland J. Kunze E. Ernestus R-I. (2021). Therapeutic hypercapnia for prevention of secondary ischemia after severe subarachnoid hemorrhage: Physiological responses to continuous hypercapnia. Sci. Rep. 11 (1), 11715. 10.1038/s41598-021-91007-7 34083595 PMC8175721

[B67] Stocchetti N. Carbonara M. Citerio G. Ercole A. Skrifvars M. B. Smielewski P. (2017). Severe traumatic brain injury: Targeted management in the intensive care unit. Lancet. Neurol. 16 (6), 452–464. 10.1016/S1474-4422(17)30118-7 28504109

[B68] Svedung Wettervik T. Engquist H. Howells T. Rostami E. Hillered L. Enblad P. (2020a). Arterial lactate in traumatic brain injury - relation to intracranial pressure dynamics, cerebral energy metabolism and clinical outcome. J. Crit. Care 60, 218–225. 10.1016/j.jcrc.2020.08.014 32882604

[B69] Svedung Wettervik T. Howells T. Hillered L. Nilsson P. Engquist H. Lewén A. (2020b). Mild hyperventilation in traumatic brain injury-relation to cerebral energy metabolism, pressure autoregulation, and clinical outcome. World Neurosurg. 133, e567–e575. 10.1016/j.wneu.2019.09.099 31561041

[B70] Svedung Wettervik T. Howells T. Ronne-Engström E. Hillered L. Lewén A. Enblad P. (2019). High arterial glucose is associated with poor pressure autoregulation, high cerebral lactate/pyruvate ratio and poor outcome following traumatic brain injury. Neurocrit. Care 31 (3), 526–533. 10.1007/s12028-019-00743-2 31123993 PMC6872512

[B71] Tasneem N. Samaniego E. A. Pieper C. Leira E. C. Adams H. P. Hasan D. (2017). Brain multimodality monitoring: A new tool in neurocritical care of comatose patients. Crit. Care Res. Pract. 2017, 6097265. 10.1155/2017/6097265 28555164 PMC5438832

[B72] Udy Andrew (2021). Brain oxygen neuromonitoring in Australia and New Zealand assessment trial. 12619001328167p [Internet]. [cited 2021 1]. Available from: https://www.anzics.com.au/current-active-endorsed-research/bonanza/.

[B73] Veldeman M. Albanna W. Weiss M. Conzen C. Schmidt T. P. Clusmann H. (2020). Treatment of delayed cerebral ischemia in good-grade subarachnoid hemorrhage: Any role for invasive neuromonitoring? Neurocrit. Care 35, 172–183. 10.1007/s12028-020-01169-x 33305337 PMC8285339

[B74] Veldeman M. Albanna W. Weiss M. Conzen C. Schmidt T. P. Schulze-Steinen H. (2020). Invasive neuromonitoring with an extended definition of delayed cerebral ischemia is associated with improved outcome after poor-grade subarachnoid hemorrhage. J. Neurosurg. 134 (5), 1527–1534. 10.3171/2020.3.JNS20375 32413866

[B75] Volovici V. Syn N. L. Ercole A. Zhao J. J. Liu N. (2022). Steps to avoid overuse and misuse of machine learning in clinical research. Nat. Med. 28, 1996–1999. 10.1038/s41591-022-01961-6 36097217

[B76] Westermaier T. Stetter C. Kunze E. Willner N. Holzmeier J. Weiland J. (2016). Controlled hypercapnia enhances cerebral blood flow and brain tissue oxygenation after aneurysmal subarachnoid hemorrhage: Results of a phase 1 study. Neurocrit. Care 25 (2), 205–214. 10.1007/s12028-016-0246-x 26886010

[B77] Winberg J. Holm I. Cederberg D. Rundgren M. Kronvall E. klund N. (2022). Cerebral microdialysis-based interventions targeting delayed cerebral ischemia following aneurysmal subarachnoid hemorrhage. Neurocrit. Care 37 (1), 255–266. 10.1007/s12028-022-01492-5 35488171 PMC9283139

[B78] Winkler M. K. Dengler N. Hecht N. Hartings J. A. Kang E. J. Major S. (2017). Oxygen availability and spreading depolarizations provide complementary prognostic information in neuromonitoring of aneurysmal subarachnoid hemorrhage patients. J. Cereb. Blood Flow. Metab. 37 (5), 1841–1856. 10.1177/0271678X16641424 27025768 PMC5435278

[B79] Yang M. T. (2020). Multimodal neurocritical monitoring. Biomed. J. 43 (3), 226–230. 10.1016/j.bj.2020.05.005 32651135 PMC7424082

[B80] Zeiler F. A. Smielewski P. Stevens A. Czosnyka M. Menon D. K. Ercole A. (2019). Non-invasive pressure reactivity index using Doppler systolic flow parameters: A pilot analysis. J. Neurotrauma 36 (5), 713–720. 10.1089/neu.2018.5987 30091677

[B81] Zhang Y. Liu X. Steiner L. Smielewski P. Feen E. Pickard J. D. (2016). Correlation between cerebral autoregulation and carbon dioxide reactivity in patients with traumatic brain injury. Acta Neurochir. Suppl. 122, 205–209. 10.1007/978-3-319-22533-3_41 27165907

