# Supplementary material for: Cerebral multimodality monitoring in adult neurocritical care patients with acute brain injury: A narrative review
Source: Front Physiol. 2022 Dec 1;13:1071161. doi: 10.3389/fphys.2022.1071161 (PMC9751622; doi:10.3389/fphys.2022.1071161)
Supplement: Supplementary file 1 [file DataSheet2.pdf]

Supplementary Material

Jeanette Tas, Marek Czosnyka, Iwan C.C. van der Horst et al. Continuous cerebral multimodality monitoring in adult neurocritical care patients with acute brain injury: a narrative review

Details of the individual cerebral multimodality (MMM) studies, including title, disease, main objective, sample size, enrollment period, monitoring modalities, and interventions.

Supplementary Table S5a | Observations - cerebral multimodality monitoring studies

Supplementary Table S5b | Systemic intervention - cerebral multimodality monitoring studies

Supplementary Table S5b | Cerebral intervention - cerebral multimodality monitoring studies

Supplementary Table S5d | Management guided - cerebral multimodality monitoring studies

Overview of individual cerebral multimodality monitoring studies

Supplementary Tables S5a, b, c, and d summarize the individual cerebral multimodality monitoring (MMM) studies (112 studies). The order of the studies is by year of publication and alphabetically by author name. Supplementary Table S5a describes the observational studies (68 studies), Supplementary Table S5b the systemic interventional studies (24 studies), Supplementary Table S5c Cerebral interventional studies* (10 studies), and Supplementary Table S5d Management guided by cerebral multimodality studies* (11 studies).
*One study was classified as both cerebral interventional and management guided by MMM.

Supplementary Table S5a | Observational cerebral multimodality monitoring studies

Ref.	Dx.	Aim or objective(s)	No. of patients (*N*)	Enrollment period	Modalities
Bouzat et al., 2015 (1)	TBI	Detect cerebral hypoperfusion using multimodality monitoring compared to unimodal (ICP) monitoring.	27	May 2010 - Nov 2013	• ICP<br>• $PbtO_2$<br>• CMD
Esnault et al., 2015 (2)	TBI (*n*=5)<br>SAH (*n*=1)<br>ICA dissection (*n*=1)<br>ICH (*n*=1)	Correlate NIRS (rSO_2) with a third-generation NIRS monitor and an invasive measure of $PbtO_2$.	8	Nov 2011 - Jan 2012	• ICP<br>• $PbtO_2$<br>• NIRS
Helbok et al., 2015 (3)	aSAH	Relate pathophysiological events involved in the development of early brain injury in poor-grade aneurysmal SAH patients over time and relate these findings to clinical course and outcome.	26	2010 - 2012	• ICP<br>• $PbtO_2$<br>• TCD[a]<br>• CMD

Ref.	Dx.	Aim or objective(s)	No. of patients (*N*)	Enrollment period	Modalities
Tackla et al., 2015 (4)	TBI	Discussing the experience in calculating a new rCBF index (correlation rCBF and CPP).	7	-	• ICP<br>• rCBF
Tholance et al., 2015 (5)	aSAH	Describe the changes in cerebral energy metabolism observed with eight hourly updated retrograde jugular vein catheter, continuous $PbtO_2$, and hourly CMD to compare these two approaches and to evaluate whether they could be complementary to predict the outcome of poor-grade aneurysmal SAH patients.	18	2006 - 2010	• $PbtO_2$<br>• TCD[a]<br>• $SvjO_2$ (update 8 hours)<br>• CMD
Budohoski et al., 2016 (6)	aSAH	Analyze the relationship between various methods of testing cerebral autoregulation and their predictive value for clinical outcome (DCI). Including combining CA-measures (TCD and NIRS).	98	Jun 2010 - Jan 2012	• TCD<br>• NIRS
Dias et al., 2016 (7)	TBI	Describe the characteristics of plateau waves with MMM results.	18	-	• ICP<br>• Cerebral T<br>• $PbtO_2$<br>• rCBF<br>• NIRS

Ref.	Dx.	Aim or objective(s)	No. of patients (*N*)	Enrollment period	Modalities
Helbok et al., 2016 (8)	ICH	Describe the incidence of SDs in a cohort of poor-grade ICH patients in whom hematoma evacuation was performed; describe the timing of ECoG SDs relative to the bleeding and perihematomal edema, and compare the ECoG curve characteristics to those patients with other etiologies of acute brain injury.	27	Jan 2013 - Jul 2015	• ICP<br>• ECoG
Hifumi et al., 2016 (9)	HIBI	Associate CMD with blood lactate and glucose levels in relation to neurological outcomes after cardiac arrest, and associate ICP with the CMD-L/P-ratio in relation to clinical outcome.	10	1 Jul 2005 - 30 Apr 2009	• ICP<br>• CMD
Hinzman et al., 2016 (10)	TBI	Examine the association of SD with changes in cerebral neurochemistry by placing a CMD probe alongside a subdural electrode strip (ECoG) in peri-lesional cortex in TBI patients requiring neurosurgery.	16	-	• ICP[a]<br>• CMD<br>• ECoG
Myers et al., 2016 (11)	TBI	Evaluate an ICP and brain tissue oxygenation prediction model and compare this model with the clinical outcome.	817	1989 - 2000<br>and 2006 - 2013	• ICP<br>• $PbtO_2$

Ref.	Dx.	Aim or objective(s)	No. of patients (N)	Enrollment period	Modalities
Papadopoulos et al., 2016 (12)	SAH	Correlate rCBF with CMD parameters in SAH patients; study the relationship with clinical outcome.	21	2009 - 2013	• ICP<br>• Cerebral T<br>• rCBF<br>• CMD
Patet et al., 2016 (13)	aSAH	Evaluate the predictive value of CMD abnormalities for the diagnosis of delayed cerebral hypoperfusion diagnosed with static brain CT-perfusion.	20	-	• ICP<br>• $PbtO_2$<br>• CMD
Vanga et al., 2016 (14)	TBI (*n* =65)<br>SAH<br>ICH<br>Other (not specified)	Examine whether $PbtO_2$, ICP, and heart rate correlated with the incidence and type of acute arrhythmias in patients with acute brain injury.	106	-	• ICP<br>• Cerebral T[a]<br>• $PbtO_2$
Vespa et al., 2016 (15)	TBI	Determine the incidence of electrographic seizures and interictal epileptiform activity on sEEG and dEEG and associate metabolic changes in CMD during interictal and ictal epileptiform discharges.	34	2009 - 2013	• ICP[a]<br>• $SvjO_2$[a]<br>• CMD<br>• sEEG<br>• dEEG

Ref.	Dx.	Aim or objective(s)	No. of patients (*N*)	Enrollment period	Modalities
Winkler et al., 2016 (16)	aSAH	Associate different MMM modalities with clinical outcome.	33	Sep 2009 - Aug 2013	• ICP<br>• $PbtO_2$<br>• Cerebral T<br>• TCD[a]<br>• ECoG
Carteron et al., 2017 (17)	aSAH	Study the relationship between CMD, $PbtO_2$, and static CBF using CT-perfusion.	18	Mar 2013 - Apr 2015	• ICP[a]<br>• $PbtO_2$<br>• CMD
Foreman et al., 2017 (18)	aSAH	Describe the relationship between rCBF and EEG (dEEG or sEEG) in both patients with and without DCI.	20	Jun 2006 - Mar 2012	• ICP<br>• $PbtO_2$ [a]<br>• rCBF<br>• sEEG<br>• dEEG
Nyholm et al., 2017 (19)	TBI	Evaluate the relationship between hyperthermia and ICP and the influence of intracranial compliance and CBF pressure autoregulation; study the relationship between hyperthermia and $PbtO_2$ and CMD.	87	Jan 2008 - Dec 2010	• ICP<br>• $PbtO_2$<br>• Cerebral T [a]<br>• CMD

Ref.	Dx.	Aim or objective(s)	No. of patients (*N*)	Enrollment period	Modalities
Pinczolits et al., 2017 (20)	AIS	Investigate whether SDs in patients with AIS are associated with altered CMD of glutamate, lactate, pyruvate, or L/P-ratio.	18	May 2009 – Apr 2011	• CMD<br>• EcoG
Schiefecker et al., 2017 (21)	ICH	Investigate the dynamics of Cerebral T relative to the occurrence of SDs and core temperature in patients with ICH.	20	Jan 2013 – Jan 2016	• ICP [a]<br>• Cerebral T<br>• EcoG
Witsch et al., 2017 (22)	SAH	Relate periodic discharges (using a change-point analysis to characterize electrophysiological changes) on sEEG with other MMM ($PbtO_2$ and rCBF) variables.	90	Jun 2006 – Sep 2014	• ICP [a]<br>• $PbtO_2$<br>• rCBF<br>• sEEG<br>• dEEG
Chen et al., 2018 (23)	ICH	Comprehensively evaluate brain function by administering TCD combined with qEEG in patients with severe acute supratentorial-ICH; assess outcome at the 90-day follow-up; explore a new basis for pathophysiological changes in severe ICH.	47	Jun 2015 – Dec 2016	• TCD<br>• sEEG

Ref.	Dx.	Aim or objective(s)	No. of patients (*N*)	Enrollment period	Modalities
Dellazizzo et al., 2018 (24)	TBI	Describe the association between PaO_2 and $PbtO_2$ to determine the minimal PaO_2 required to maintain $PbtO_2$; investigate the relationship with clinical outcome.	38	2013 - 2016	• ICP<br>• $PbtO_2$
Dreier et al., 2018 (25)	TBI (*n*=5)<br>aSAH (*n*=3)<br>AIS (*n*=1)	Analyze pathological events in patients during abrupt hypoxia-ischemia after withdrawal from life-sustaining treatments.	9	-	• ICP (*n*=9)<br>• $PbtO_2$ (*n*=6)<br>• rCBF (LDF) (*n*=2)<br>• Cerebral T (*n*= 4)<br>• ECoG (*n*=4)<br>• dEEG (*n*=5)<br>• sEEG (*n*=1)
Foreman et al., 2018 (26)	TBI	Describe the safety and the reliability of a single burr hole access for invasive MMM monitoring application.	43	Mar 2015 - Mar 2017	• ICP<br>• $PbtO_2$<br>• Cerebral T<br>• rCBF<br>• dEEG
Lückl et al., 2018 (27)	aSAH	Characterize negative ultraslow potential (NUP). First party of the study is an animal study. Second part was a clinical study using simultaneous DC/AC-ECoG, $PbtO_2$, rCBF, ICP, and ABP recordings; serial neuroimaging scans were used to determine whether electrodes displaying the NUP are more likely to overlie a newly developing ischaemic lesion than electrodes not displaying the NUP.	11	-	• ICP (*n*=1)<br>• $PbtO_2$ (*n*=8)<br>• Cerebral T[a]<br>• rCBF [a] (LDF) (*n*=4)<br>• ECoG (*n*=1)

**Ref.**	**Dx.**	**Aim or objective(s)**	**No. of patients (*N*)**	**Enrollment period**	**Modalities**
Morris et al., 2018 (28)	aSAH + HIBI (*n*=31)<br>aSAH (*n*=146)	Describe the differences in MMM results between SAH with and without additional cerebral hypoxia at presentation. Three hypotheses were tested: (1) clinical phenotypes between the groups differ and may suggest the cause of arrest, (2) brain physiology measures would demonstrate cerebrovascular decompensation in the HIBI patients despite identical CPP and management targets, (3) outcomes of aggressively treated HIBI patients should not significantly differ from those of non-HIBI patients.	177	Jul 1996 - Jun 2016	• ICP<br>• $PbtO_2$<br>• rCBF<br>• CMD<br>• sEEG
Alkhachroum et al., 2019 (29)	SAH	Evaluate the relationship between CPP, CPPopt, deltaCPP (calculated as CPP-CPPopt), and PRx during seizures and ictal-interictal continuum.	73	Jun 2006 - Jun 2013	• ICP<br>• $PbtO_2$<br>• dEEG
Bailey et al., 2019 (30)	• TBI (*n*=286)<br>• SAH (*n*=133)<br>• ICH (*n*=25)<br>• Penetrating TBI (*n*=20)<br>• Other (*n*=37)[b]	Describe the safety of a triple-lumen bolt placement by reporting the number of placed devices and the post-insertion complications.	501	8 year	• ICP<br>• $PbtO_2$<br>• Cerebral T

Ref.	Dx.	Aim or objective(s)	No. of patients (*N*)	Enrollment period	Modalities
Calviello et al., 2019 (31)	TBI	Study the feasibility of methods most suitable for describing patient cerebral hemodynamics; build a function to monitor changes in intracranial compliance, compare invasive and non-invasive calculations, and compare data-driven trend charts.	52	1992 – 2012	• ICP<br>• TCD
Davie et al., 2019 (32)	TBI	Determine the incidence and severity of rSO_2 desaturation in patients with TBI and determine the feasibility of monitoring rSO_2 in the ICU; examine the correlation between rSO_2 and ICP and rSO_2 and MAP.	20	Jul 2016 – Sep 2017	• ICP<br>• NIRS
Donnelly et al., 2019 (33)	TBI	Describe cerebral oxygenation and cerebral autoregulation indices PRx, and relate AMP (ICP amplitude) response to severe and sustained intracranial hypertension.	33	1992 – 2017	• ICP<br>• $PbtO_2$
Forsse et al., 2019 (34)	aSAH	Describe the association between CMD and jugular bulb microdialysis (JBMD); correlate CMD abnormalities with clinical outcome.	12	Sep 2017 – Mar 2018	• ICP<br>• $PbtO_2$<br>• CMD<br>• JBMD [a]

Ref.	Dx.	Aim or objective(s)	No. of patients (*N*)	Enrollment period	Modalities
Launey et al., 2019 (35)	TBI (*n*=68)<br>Healthy control (*n*=27)	Characterize cerebrovascular physiology within brain regions that initially appear structurally normal; describing temporal changes in physiology, changes in flow-metabolism, local microvascular flow-volume association, disentangle ischemia from coupled hypoperfusion and assess the contribution of vascular engorgement to ICP elevation.	68	Feb 1998 - 2014	• ICP<br>• $PbtO_2$<br>• $SvjO_2$
Rajagopalan et al., 2019 (36)	HIBI (*n*=6)<br>TBI (*n*=3)<br>SAH (*n*=2)	Identify and describe trends in ICP, CPP, PRx, $PbtO_2$, rCBF, Cerebral T, and CMD that occur during progression to and at the time of brain death.	11	Oct 2015 - Jun 2018	• ICP<br>• $PbtO_2$<br>• Cerebral T<br>• rCBF<br>• CMD
Silverman et al., 2019 (37)	aSAH	Describe the feasibility of invasive (ICP, PRx, CPPopt) and non-invasive (NIRS, TOx, ABPopt) CA measures; correlate invasive and non-invasive results, including the autoregulation limits; relate deviation from CPPopt/ABPopt with radiographic and clinical outcome.	31	-	• ICP<br>• TCD [a]<br>• NIRS
Wettervik et al., 2019 (38)	TBI	Investigate the association between arterial glucose, PRx, and CMD	120	2008 - 2018	• ICP<br>• CMD

Ref.	Dx.	Aim or objective(s)	No. of patients (*N*)	Enrollment period	Modalities
Addis et al., 2020 (39)	aSAH	Describe the relationship between delta T (cerebral T – T systemic) and (preserved) brain metabolic activity; describe the relationship between delta T and clinical outcome.	46	2010 - 2016	• ICP<br>• Cerebral T<br>• rCBF<br>• CMD
Gagnon et al., 2020 (40)	TBI (*n*=20)<br>ICH (*n*=5)	Describe the frequency of hypoxic episodes and their characteristics.	25	Jun 2017 - Aug 2018	• ICP<br>• $PbtO_2$<br>• Cerebral T
Marini et al., 2020 (41)	TBI	Evaluate the relationship between CPP and estimates of cerebral oxygenation (NIRS, $PbtO_2$) and CMD.	20	-	• ICP<br>• $PbtO_2$<br>• CMD<br>• NIRS
Rass et al., 2020 (42)	TBI	Quantify changes in $PbtO_2$ during temperature increases in severe TBI to explore simultaneous changes of hemodynamic parameters and the CA state; compare periods of temperature increases identified through visual plot analysis and by algorithm-supported detection.	33	Jan 2015 - Dec 2017	• ICP<br>• $PbtO_2$<br>• Cerebral T (*n*=16)

Ref.	Dx.	Aim or objective(s)	No. of patients (*N*)	Enrollment period	Modalities
Robba et al., 2020 (43)	TBI	Indicating the threshold of cerebral hypoxia and parameters that are determinants of cerebral hypoxia; assess whether any of these indices ($PbtO_2$, PaO_2, $PbtO_2/PaO_2$, ratio of $PbtO_2$ to FiO_2, and PaO_2/FiO_2) based on the relationship between cerebral tissue oxygenation and systemic arterial oxygenation and the fraction of inspired oxygen, can be helpful for prognostication of mortality in TBI patients.	70	Nov 2014 – Oct 2018	• ICP<br>• $PbtO_2$
Sekhon et al., 2020 (44)	HIBI	Characterizes the difference in the diffusion gradient (Pvo_2 – $PbtO_2$) of cellular oxygen delivery and the presence of diffusion limitation physiology (the relationship between the Pvo_2 – $Pbto_2$ gradient and CPP) in hypoxic-ischemic brain injury patients with brain hypoxia ($PbtO_2 < 20$ mm Hg) versus normoxia ($PbtO_2 > 20$ mm Hg).	14	Nov 2016 – Jan 2019	• ICP<br>• $PbtO_2$<br>• $SvjO_2$
Wettervik et al., 2020 (45)	TBI	Clarify the association on endogenous arterial lactate in relation to intracranial pressure dynamics, pressure CBF autoregulation, cerebral energy metabolism, systemic injuries, and clinical outcome following severe TBI.	115	2008 – 2018	• ICP<br>• CMD

Ref.	Dx.	Aim or objective(s)	No. of patients (N)	Enrollment period	Modalities
Zeiler et al., 2020 (46)	TBI	Explore the relationship between insults in ICP, $PbtO_2$, and PRx and investigate preliminary associations with outcome. Insults in the variables were studied by: (1) % of time with ICP > 20 mmHg (2) % of time with $PbtO_2$ < 20 mmHg (3) % of time with ICP PRx > threshold (0.25, 0.35 were used) (4) % of time with normal or abnormal ICP PRx and normal or abnormal $PbtO_2$ (<20 or >20 mmHg).	43	Jan 2015 - Dec 2017	• ICP<br>• $PbtO_2$
Zeiler et al., 2020 (47)	TBI	Study the physiological consequences of high versus normal ICP periods by comparing % of time (and dose) above or below thresholds for ICP, $PbtO_2$, and CA index PRx.	185	Jan. 2015 - Dec 2017	• ICP<br>• $PbtO_2$
Zeiler et al., 2020 (48)	TBI	Explore the relationship between slow-wave fluctuations in ICP, MAP, and $PbtO_2$ over time.	47	Jan 2015 - Dec 2017	• ICP<br>• $PbtO_2$
Balu et al., 2021 (49)	HIBI<br><br>Other (hypoxic injury without cardiac arrest)	Describe the clinical characteristics of monitored patients; evaluate the feasibility and complication rate of invasive neuromonitoring; assess the association of intracranial neuromonitoring parameters with neurological outcome.	36	Oct 2015 - Jun 2018	• ICP<br>• $PbtO_2$

Ref.	Dx.	Aim or objective(s)	No. of patients (*N*)	Enrollment period	Modalities
Barajraji et al., 2021 (50)	SAH (*n*=56)<br>TBI (*n*=35)<br>ICH (*n*=200)<br>Medical, not specified (*n*=2)	Evaluate a single-center experience of an intracranial multimodal monitoring bolt system regarding surgical placement and related complications, management, technical malfunctions, and adverse events.	113	Jul 2016 - Jan 2020	• ICP<br>• $PbtO_2$<br>• CMD<br>• dEEG
Birg et al., 2021 (51)	TBI	Describe Cerebral T, ICP, and CPP; clarify the relationship between Cerebral T, ICP, and CPP during Cerebral T changes.	21	Jan 2015 - Dec 2017	• ICP<br>• Cerebral T<br>• $PbtO_2$[a]
Guilfoyle et al., 2021 (52)	TBI	Quantify the independent effect of L/P-ratio and cerebral glucose on neurological outcome; characterize the temporal course of these parameters following TBI; assess the functional relationship between energy metabolism and other monitoring variables to inform an outline clinical protocol for managing traumatic, metabolic function.	619	1997 - 2016	• ICP<br>• $PbtO_2$<br>• CMD
Hoiland et al., 2021 (53)	HIBI (*n*=18)<br>Healthy (*n*=14)	Assess brain hypoxia ($PbtO_2$<20) versus no brain hypoxia ($PbtO_2$>20) regarding elevated biomarkers (neuronal, astroglial, endothelial injury) and inflammation; assess if brain hypoxia is related to a diffusion limitation of O_2 and that hyperosmolar therapy reduces brain hypoxia.	18	2016 - 2019	• ICP<br>• $PbtO_2$<br>• JVP [a]

Ref.	Dx.	Aim or objective(s)	No. of patients (*N*)	Enrollment period	Modalities
Kieninger et al., 2021 (54)	aSAH (*n*=22)<br><br>Carotid artery aneurysm (*n*=4)	Describe complications related to MMM; describe therapeutic measures derived in cases of pathological values in MMM.	26	Jan 2012 - Dec 2017	• ICP<br>• $PbtO_2$<br>• rCBF<br>• TCD [a]<br>• CMD<br>• NIRS
Kuo et al., 2021 (55)	TBI	Characterize the cerebral T rhythm and investigate its prognostic value in terms of postoperative mortality and functional outcome in patients with moderate to severe TBI.	108	May 2011 - Dec 2017	• ICP<br>• Cerebral T
Lindner et al., 2021 (56)	ICH	Define ABP targets in poor-grade ICH patients during postoperative care after hematoma evacuation based on the lowest prevalence of brain tissue hypoxia and metabolic distress.	40	2011 - 2018	• ICP<br>• $PbtO_2$<br>• CMD
Mueller et al., 2021 (57)	SAH	Compare the ability of qEEG and TCD/TCCS to provide early identification of cerebral infarction on imaging.	34	Nov 2011 - Feb 2013<br>and<br>Nov 2014 - May 2016	• TCD or TCCS<br>• sEEG
Qi et al., 2021 (58)	AIS	Explore the possibility of obtaining more accurate and comprehensive prognostic predictors for patients with large hemispheric infarction by TCD-qEEG.	59	Jul 2018 - Dec 2019	• TCD<br>• sEEG

Ref.	Dx.	Aim or objective(s)	No. of patients (*N*)	Enrollment period	Modalities
Rajagopalan et al., 2021 (59)	TBI (*n*=6)<br><br>aSAH (*n*=14)	Explore a data-driven approach to group invasive MMM measurements to identify distinct physiological states that inform brain injury mechanisms and outcome prediction.	20	Mar 2005 - Sep 2009	• ICP<br>• $PbtO_2$<br>• CMD
Schumm et al., 2021 (60)	AIS	Investigate the effect of physiological variables (body temperature, MAP, CPP, and ICP) on the incidence and features of SD in the postoperative monitoring period following hemicraniectomy.	60	Aug 2008 - Mar 2017	• ICP<br>• ECoG
Wettervik et al., 2021 (61)	TBI	Investigate the impact of CPP insults (according to CPPopt thresholds and Brain Trauma Foundation thresholds) on CMD results and clinical outcome.	98	2008 – 2018	• ICP<br>• CMD
Wettervik et al., 2021 (62)	TBI	Elucidate the role of arterial oxygenation, the incidence of hypoxia, hyperoxia, and the relation to cerebral metabolism, cerebrovascular reactivity, and clinical outcome.	115	2008 – 2018	• ICP<br>• CMD
Wettervik et al., 2021 (63)	TBI	Determine the incidence and temporal course of hyperthermia after TBI and its relation to ICP dynamics and cerebral energy metabolism; determine whether hyperthermia was associated with clinical outcomes following TBI.	115	2008 - 2018	• ICP<br>• CMD

Ref.	Dx.	Aim or objective(s)	No. of patients (*N*)	Enrollment period	Modalities
Dreier et al., 2022 (64)	aSAH	Investigate whether the peak total SD-induced depression duration of a recording day during delayed neuromonitoring (delayed SD duration) indicates delayed ipsilateral infarction. Secondary outcome measures: occurrence of delayed neurological deficit in clinically assessable, non-sedated patients; manually segmented volumes of ipsilateral damage due to ICH, ECI, and DCI alone or in combination; MRI segmentation analysis; clinical outcome analysis; multivariate analysis including neuromonitoring data and digital subtraction angiography.	180	Sep 2009 - Apr 2018	• ICP (*n*=150)<br>• $PbtO_2$ (*n*=71) [a]<br>• rCBF (LDF) (*n*=22) [a]<br>• TCD (*n*=157)<br>• ECoG
Owen et al., 2022 (65)	aSAH	Elucidate the relationship between various CA measures and assess how they relate to both clinical outcomes and SD incidence.	19	-	• ICP<br>• $PbtO_2$<br>• rCBF<br>• NIRS<br>• ECoG
Wettervik et al., 2022 (66)	aSAH	Investigate the association among the arterial metabolic content variables PaO_2, $PaCO_2$, glucose, and lactate with the PRx and energy metabolism; study the relationship between delayed ischemic neurological deficits (DIND) and clinical outcome.	60	Nov 2016 - May 2021	• ICP<br>• CMD

Ref.	Dx.	Aim or objective(s)	No. of patients (*N*)	Enrollment period	Modalities
Wettervik et al., 2022 (67)	aSAH	Determine the association between ICP- and CPP threshold insults and cerebral energy metabolism; determine the association of these insults with clinical outcome.	75	2008 - 2018	• ICP<br>• CMD
Yang et al., 2022 (68)	TBI (*n* =102)<br><br>Healthy (*n*=55)	Evaluate the effect of the apolipoprotein E (*APOE*) *ε4* allele on rSO_2 and qEEG at the early stage of TBI and explore the relationship between APOE and cerebral oxygen saturation and brain electrical activity.	102	Sep 2018 - Sep 2020	• sEEG<br>• NIRS

[a] Other modalities. Modalities described in the study but not part of the study aim or objective(s). These modalities were either used for clinical management or not part of our included modalities, but continuous or daily updated monitoring.
[b] Other diseases included: cerebral arteriovenous malformation, intracranial infections, hepatic encephalopathy, and cerebral edema from ornithine transcarbamylase deficiency.
ABP = arterial blood pressure; ABPopt = optimal arterial blood pressure; AIS = acute ischemic stroke; CA = cerebral autoregulation; Cerebral T = cerebral temperature; CMD = cerebral microdialysis; CPP = cerebral perfusion pressure; CPPopt = optimal cerebral perfusion pressure; CT = computer tomography; DC/AC = Direct current /alternate current (AC)-electrocorticography; DCI = diffuse cerebral ischemia; dEEG = depth electroencephalography; DIND = delayed ischemic neurological deficits; ECI = early cerebral ischaemia; ECoG = electrocorticography; FiO_2 = fraction of inspired oxygen; HIBI: hypoxic ischemic brain injury following cardiac arrest; ICA = internal carotid artery; ICH = intracerebral hemorrhage; ICP = intracranial pressure; ICU = intensive care unit; JBMD = jugular bulb microdialysis; JVP = jugular venous pressure; L/P-ratio = lactate/pyruvate ratio; LDF = Laser Doppler flowmetry; MAP = mean arterial blood pressure; MRI = magnetic Resonance Imaging; NIRS = near-infrared spectroscopy; No. = number; PaO_2 = partial arterial oxygenation pressure; PaO_2 = partial pressure of oxygen; $PbtO_2$ = partial pressure of brain tissue oxygenation; PRx = pressure reactivity index; rCBF = regional cerebral blood flow; (a)SAH = (aneurysmal)subarachnoid hemorrhage; SD = spreading depolarisation; sEEG = surface electroencephalography; $SvjO_2$ = jugular bulb venous oximetry; TBI = traumatic brain injury; TCCS = transcranial colour doppler sonography; TCD = transcranial Doppler; TOx = tissue oxygenation index.

Supplementary Table S5b | Systemic interventions - cerebral multimodality monitoring studies

Ref.	Dx.	Aim or objective(s)	No. of patients (*N*)	Enrollment period	Intervention	Modalities
Flynn et al. 2015 (69)	TBI	Examine the effect of induction of therapeutic hypothermia on ICP and $PbtO_2$.	17	-	Hypothermia	• ICP<br>• $PbtO_2$<br>• Cerebral T[a]
Kurtz et al. 2015 (70)	aSAH	Investigate the effect of packed red blood cell (RBC) transfusion on cerebral oxygenation and metabolism.	15	Jan 2008 - Jun 2009	RBC transfusion	• ICP [a]<br>• $PbtO_2$<br>• CMD
Sekhon et al. 2015 (71)	TBI	Study the effect of packed RBC-transfusion on the CA index PRx. Primary outcome was the change in CA, as measured by PRx, after RBC transfusion. Secondary outcomes: the change in hemoglobin concentration and $PbtO_2$ after RBC transfusion.	28	Jan 2007 - 30 Jun 2014	RBC transfusion	• ICP<br>• $PbtO_2$

Ref.	Dx.	Aim or objective(s)	No. of patients (*N*)	Enrollment period	Intervention	Modalities
Ghosh et al. 2016 (72)	TBI *n*=7)<br><br>SAH (*n*=8)<br><br>ICH (*n*=1)	Investigate the oxygen dependence (NIRS, $PbtO_2$, TCD) of mitochondrial metabolism (CMD L/P-ratio) in vivo following acute brain injury.	16	-	Hyperoxia	• $PbtO_2$<br>• TCD<br>• CMD<br>• NIRS
McCredie et al. 2016 (73)	TBI	Study the effect of packed RBC transfusion on NIRS rSO_2; correlate rSO_2 changes to other systemic- and cerebral variables; study the dependence of rSO_2 on baseline hemoglobin level; study the effect on fractional tissue oxygen extraction.	19	Nov 2012 - Mar 2014	RBC transfusion	• ICP<br>• NIRS
Sahoo et al. 2016 (74)	TBI	Evaluate the effect of normobaric hyperoxia on CBF velocity (TCD) and NIRS in operated TBI patients. In addition, study the effect on CA status.	50	-	Hyperoxia	• TCD<br>• NIRS
Westermaier et al. 2016 (75)	SAH	Investigate changes in rCBF during hypercapnia, and compare rCBF with TCD mean CBFV and NIRS rSO_2 results.	12	Jan 2013 - Feb 2014	Hypercapnia	• ICP<br>• Cerebral T [a]<br>• rCBF<br>• NIRS<br>• TCD

Ref.	Dx.	Aim or objective(s)	No. of patients (*N*)	Enrollment period	Intervention	Modalities
Zhang et al. 2016 (76)	TBI	Correlate cerebral autoregulation indices (TCD-based) ARI and Mx with carbon dioxide reactivity during transient mild hypocapnia (hyperventilation period of 60 min).	31	-	Hypocapnia	• ICP<br>• TCD
Kofler et al. 2017 (77)	SAH	Investigate the effect of enteral nutrition on CMD and ICP.	17	2010 - 2012	Enteral nutrition	• ICP<br>• Cerebral T[a]<br>• CMD
Carteron et al. 2018 (78)	TBI (*n*=13)<br><br>aSAH (*n*=10)	Analyze changes - compared with baseline TCD-derived indexes of cerebral perfusion and brain extracellular concentrations of energy metabolites (CMD lactate and glucose) during a 3-hour intravenous infusion of hypertonic lactate, administered as a resuscitation fluid during the early post-injury phase + clinical outcome.	23	Mar 2012 - Dec 2017	Hypertonic lactate	• ICP[a]<br>• TCD<br>• CMD
Jakkula et al. 2018 (79)	HIBI	Compare results between patients treated with low (65-75 mmHg) or high (80-100 mmHg) MAP values: evaluate serum biomarkers (like NSE) at 48 hr after cardiac arrest to study the regional differences in frontal NIRS-based rSO2% and compare sEEG findings.	120	22 Mar 2016 -<br>3 Nov 2017	ABP-management	• NIRS<br>• sEEG

Ref.	Dx.	Aim or objective(s)	No. of patients (*N*)	Enrollment period	Intervention	Modalities
Brandi et al. 2019 (80)	TBI	Study the (adverse) effects of moderate short-term hyperventilation during the acute phase on cerebral hemodynamics (ICP/CPP), oxygenation ($PbtO_2$), and metabolism (CMD).	11	May 2014 - May 2017	Hyperventilation	• ICP<br>• $PbtO_2$<br>• TCD (TCCD)<br>• CMD
Sekhon et al. 2019 (81)	HIBI	Describe the relationship between $PbtO_2$ and MAP over time using a tier-based clinical protocol to manage increased ICP in combination with low $PbtO_2$. Other endpoints included the observed PRx, $SvjO_2$, and rSO_2 values over time.	10	Nov 2016 - Jan 2018	ABP-management	• ICP<br>• $PbtO_2$<br>• $SvjO_2$<br>• NIRS
Wettervik et al. 2020 (82)	TBI	Evaluate the safety and treatment effect of mild hyperventilation (4.0 - 4.5 kPa/30-34 mm Hg) in TBI; evaluate mild hyperventilation and its effect on ICP and CMD, pressure autoregulation, and clinical outcome.	120	2008 - 2018	Hyperventilation	• ICP<br>• CMD
Burnol et al. 2021 (83)	TBI (*n*=16)<br><br>SAH (*n*=3)<br><br>AIS (*n*=3)<br><br>Other, not specified (*n*=1)	Study the effect of a 30° upright posture versus 15° versus 0° on brain oxygenation and circulation; sub-analysis of patients who underwent a decompressive craniectomy to investigate whether changes in brain compliance could affect these potential effects.	23	Feb 2012 - Sep 2015	Head-bed posture	• ICP<br>• $PbtO_2$<br>• TCD

Ref.	Dx.	Aim or objective(s)	No. of patients (*N*)	Enrollment period	Intervention	Modalities
Dagod et al. 2021 (84)	TBI	Assess the impact of the temporary changeover from the half-seated position to the lying position on CPP and oxygenation parameters during the first week of management of patients admitted for severe TBI.	24	Sep 2014 - Oct 2016	Head-bed posture	• ICP<br>• TCD<br>• $SvJO_2$<br>• NIRS
Hosmann et al. 2021 (85)	aSAH	Study the impact of intrahospital transport to the CT-scanner on MMM in patients suffering SAH, with a particular focus on cerebral metabolism.	20	-	Intrahospital transport	• ICP<br>• $PbtO_2$<br>• Cerebral T<br>• TCD<br>• CMD
Kovacs et al. 2021 (86)	TBI (*n*=20)<br><br>SAH (*n*=29)<br><br>ICH (*n*=4)	Evaluate which level of CPP corresponds to an adequate $PbtO_2$ in a heterogeneous population of brain-injured patients; assess whether this level of CPP changes over time and characterize patients requiring higher than recommended CPP targets to have optimal brain oxygenation values.	53	Jan 2016 - Dec 2020	ABP-management	• ICP<br>• $PbtO_2$<br>• TCD<br>• NeurOptics [a] (neurological pupil index)
Rass et al. 2021 (87)	SAH	Investigate the effect of fluid management and cardiac function on $PbtO_2$ by integrating advanced hemodynamic and invasive neuromonitoring tools.	60	2010 - 2019	Fluid management	• ICP<br>• $PbtO_2$

Ref.	Dx.	Aim or objective(s)	No. of patients (*N*)	Enrollment period	Intervention	Modalities
Stetter et al. 2021 (88)	aSAH	Investigate the course of rCBF (primary) and rSO_2 (secondary) during longer-term hypercapnia and determine the time point at which physiologic adaptation mechanisms start to mitigate the CBF-increasing effect in order to locate the optimum duration of controlled hypercapnia in poor-grade SAH patients.	12	Jan 2015 - Jun 2017	Hypercapnia	• ICP<br>• rCBF<br>• TCD<br>• NIRS
Bernini et al. 2022 (89)	TBI (*n*=13)<br><br>SAH (*n*=4)	Study patients who received sequential osmotherapy with hypertonic saline (HS) followed by hypertonic lactate (HL) and compare HL with HS by studying the effect on ICP, $PbtO_2$ and CMD, ABP, pH, and chloride.	17	Nov 2016 - Dec 2019	Hypertonic saline and hypertonic lactate	• ICP<br>• $PbtO_2$<br>• CMD
Bogossian et al. 2022 (90)	TBI (*n*=21)<br><br>SAH (*n*=42)<br><br>ICH (*n*=6)	Identify factors associated with $PbtO_2$ changes after RBC transfusion in a heterogeneous population of brain-injured patients.	69	2012 - 2020	RBC transfusion	• ICP<br>• $PbtO_2$

Ref.	Dx.	Aim or objective(s)	No. of patients (*N*)	Enrollment period	Intervention	Modalities
Gargadennec et al. 2022 (91)	TBI (*n*= 17)<br><br>SAH (*n* = 29)<br><br>ICH (*n*= 7)	Compare the accuracy of ICP, $PbtO_2$, or oxygen ratio (OxR) and their combinations to detect cerebral hypoperfusion in brain-injured patients; the accuracy to detect cerebral hypoperfusion of all these variables according to the underlying brain disease.	53	Jan 2016 - Aug 2019	Hyperoxia	• ICP<br>• $PbtO_2$<br>• CMD<br>• dEEG
Hosmann et al. 2022 (92)	SAH	Evaluate whether a quick and easy-to-perform hyperoxic challenge of 3-minutes can identify patients at risk for cerebral ischemia detected by CMD.	20	Feb 2016 - Jan 2019	Hyperoxia	• ICP<br>• $PbtO_2$<br>• TCD<br>• CMD

[a] Other modalities. Modalities described in the study but not part of the study aim/objective(s). These modalities were either used for clinical management or not part of our included modalities, but continuous or daily updated monitoring.

ABP = arterial blood pressure; ABPopt = optimal arterial blood pressure; AIS = acute ischemic stroke; ARI = autoregulation index; CA = cerebral autoregulation; CBFV = cerebral blood flow velocity; Cerebral T = cerebral temperature; CMD = cerebral microdialysis; CPP = cerebral perfusion pressure; CPPopt = optimal cerebral perfusion pressure; CSF = cerebral spinal fluid; CT = computer tomography; DCI = diffuse cerebral ischemia; dEEG = depth electroencephalography; ECoG = electrocorticography; FiO_2 = fraction of inspired oxygen; HL = hypertonic lactate; HS = hypertonic saline; HIBI: hypoxic ischemic brain injury following cardiac arrest; ICH = intracerebral hemorrhage; ICP = intracranial pressure; L/P-ratio = lactate/pyruvate ratio; MAP = mean arterial blood pressure; Mx = correlation between CPP and cerebral blood flow velocity; NIRS = near-infrared spectroscopy; No. = number; OxR = oxygen ratio; PaO_2 = partial arterial oxygenation pressure; PaO_2 = partial pressure of oxygen; $PbtO_2$ = partial pressure of brain tissue oxygenation; PRx = pressure reactivity index; RBC = red blood cell; rCBF = regional cerebral blood flow; (a)SAH = (aneurysmal) subarachnoid hemorrhage; SD = spreading depolarization; sEEG = surface electroencephalography; $SvjO_2$ = jugular bulb venous oximetry; TBI = traumatic brain injury; TCD = transcranial Doppler.

Supplementary Table S5c | Cerebral intervention - cerebral multimodality monitoring studies

Ref.	Dx.	Aim or objective(s)	No. of patients (N)	Enrollment period	Intervention	Modalities
Hockel et al., 2015 (93)	aSAH	Evaluate the feasibility and safety of long-term continuous intra-arterial nimodipine (CIAN) therapy; test the effects on macrovascular vasospasm, $PbtO_2$, and cerebrovascular autoregulation indices, including PRx and ORx; evaluate 3-months clinical outcome.	13	10 months	Nimodipine	• ICP<br>• $PbtO_2$<br>• Cerebral T[a]<br>• TCD
Akbik et al., 2017 (94)	TBI	Study the effect of CSF drainage on $PbtO_2$ and parenchymal ICP.	40	2014 - 2016	CSF drainage	• ICP<br>• $PbtO_2$
Albanna et al., 2017 (95)	aSAH	Investigate the effect of endovascular rescue therapy (angioplasty /intraarterial lysis) for refractory vasospasm by $PbtO_2$ and CMD results.	13	Dec 2014 - 2015	Endovascular rescue therapies	• $PbtO_2$<br>• CMD<br>• TCD [a]
Hockel et al., 2017 (96)	aSAH	Determine the effect of different modes of nimodipine application on vasoreactivity as assessed by PRx, with particular focus on the temporal profile after intra-arterial bolus application and continuous infusion and concomitant changes in cerebral oxygen pressure as a surrogate marker of CBF.	105	2009 - Jul 2015	Nimodipine	• ICP<br>• $PbtO_2$<br>• Cerebral T [a]<br>• TCD

Ref.	Dx.	Aim or objective(s)	No. of patients (N)	Enrollment period	Intervention	Modalities
Lubillo et al., 2018 (97)	TBI	Investigate changes in $PbtO_2$ both before- and after decompressive craniectomy; and determine whether these changes could be used as an independent prognostic factor in patients with severe TBI and refractory ICH without an indication for intracranial mass lesion evacuation.	42	2002 - 2014	Decompressive craniectomy	• ICP<br>• $PbtO_2$
Ding et al., 2019 (98)	aSAH	Study the relationship between neuroglobin on CMD and $PbtO_2$ and clinical outcome at 12 months.	36	Jan 2017 - 2018	Neuroglobin	• ICP [a]<br>• $PbtO_2$<br>• TCD [a]<br>• CMD
Hosmann et al., 2019 (99)	aSAH	Investigate whether papaverine-hydrochloride mediated resolution of angiographic vasospasm translates into improved measures of CMD and oxygenations.	10	-	Papaverine-Hydrochloride	• ICP [a]<br>• $PbtO_2$<br>• TCD<br>• CMD
Ianosi et al., 2019 (100)	aSAH	Describe the effects of intravenous paracetamol, metamizole, and diclofenac during febrile and nonfebrile episodes on brain and systemic temperature and hemodynamics.	77	Apr 2010 - Jul 2017	Analgesia	• ICP<br>• $PbtO_2$<br>• Cerebral T

Ref.	Dx.	Aim or objective(s)	No. of patients (N)	Enrollment period	Intervention	Modalities
Koskinen et al., 2019 (101)	TBI	Study the effect of prostacyclin or placebo treatment on the cerebrovascular pressure reactivity and CMD glycerol marker; study the relationship between glycerol and the cerebrovascular pressure reactivity.	45	Jan 2002 - Dec 2005	Prostacyclin	• ICP<br>• CMD-glycerol
Khellaf et al., 2021 (102)	TBI	Assess the feasibility and utility of a systematic protocol to identify, characterize and treat derangements of cerebral microdialysis-derived L/P-ratio >25 within MMM; target a novel focally administered intervention, succinate in patients identified as having mitochondrial derangement based on MMM parameters.	33	Sep 2017 - Jun 2019	Succinate	• ICP<br>• $PbtO_2$<br>• CMD

[a] Other modalities. Modalities described in the study but not part of the study aim/objective(s). These modalities were either used for clinical management or not part of our included modalities, but continuous or daily updated monitor.

ABP = arterial blood pressure; ABPopt = optimal arterial blood pressure; AIS = acute ischemic stroke; CA = cerebral autoregulation; CBFV = cerebral blood flow velocity; Cerebral T = cerebral temperature; CIAN = Continuous intra-arterial nimodipine; CMD = cerebral microdialysis; CPP = cerebral perfusion pressure; CPPopt = optimal cerebral perfusion pressure; CSF = cerebral spinal fluid; CT = computer tomography; DCI = diffuse cerebral ischemia; dEEG = depth electroencephalography; ECoG = electrocorticography; FiO_2 = fraction of inspired oxygen; HIBI: hypoxic ischemic brain injury following cardiac arrest; ICH = intracerebral hemorrhage; ICP = intracranial pressure; L/P-ratio = lactate/pyruvate ratio; MAP = mean arterial blood pressure; NIRS = near-infrared spectroscopy; NSE = Neuron-Specific Enolase; No. = number; OxR = oxygen ratio; PaO_2 = partial arterial oxygenation pressure; PaO_2 = partial pressure of oxygen; $PbtO_2$ = partial pressure of brain tissue oxygenation; PRx = pressure reactivity index; RBC = red blood cell; rCBF = regional cerebral blood flow; (a) SAH = (aneurysmal) subarachnoid hemorrhage; SD = spreading depolarization; sEEG = surface electroencephalography; $SvjO_2$ = jugular bulb venous oximetry; TBI = traumatic brain injury; TCD = transcranial Doppler.

Supplementary Table S5d | Management-guided - cerebral multimodality monitoring studies

Ref.	Dx.	Aim or objective(s)	No. of patients (N)	Enrollment period	Modalities
Bele et al., 2015 (103)	aSAH	Feasibility, patient selection, and overall outcome by applying a treatment protocol for CIAN in patients with refractory cerebral vasospasm.	41	Jan 2008 - Aug 2014	• ICP<br>• $PbtO_2$<br>• rCBF<br>• TCD
Min Lin et al., 2015 (104)	TBI	Study the effect of $PbtO_2$-guided therapy compared to ICP-guided therapy on the management of cerebral variables, therapeutic interventions, survival rates, and neurological outcome.	50	Jan 2009 - Dec 2010	• ICP<br>• $PbtO_2$
Okonkwo et al., 2017 (105)	TBI	Study the efficacy and safety of an ICP + $PbtO_2$ treatment protocol. Differences in outcome between ICP+$PbtO_2$ protocol compared to the ICP-only treatment protocol.	119	Oct 2009 - Mar 2014	• ICP<br>• $PbtO_2$
Rass et al., 2019 (106)	SAH	Describe the incidence of brain tissue hypoxia burden (% of the time with $PbtO_2 < 20$ mmHg) when a strict $PbtO_2$-guided protocol is applied and describe which factors are related to brain tissue hypoxia.	100	2010 - 2017	• ICP<br>• $PbtO_2$<br>• CMD [a]

Ref.	Dx.	Aim or objective(s)	No. of patients (N)	Enrollment period	Modalities
Sekhon et al., 2017 (107)	TBI	Examine the impact of implementing a dedicated NICU program, consisting of a collaborative model of care amongst the consulting neurosurgeon and neurointensivist with a primary care attending intensivist in a general mixed medical-surgical ICU on the long-term outcome; assessing the effect of NICU on the process of care metrics, i.e., neurophysiological and temperature management.	113	Feb 2010 - May 2016	• ICP<br>• $PbtO_2$<br>• $SvjO_2$ [a]
Veldeman et al., 2020 (108)	aSAH	Assess the impact of invasive neuromonitoring on the clinical course and clinical outcome of good-grade SAH patients who experience secondary deterioration.	54	2010 - 2018	• ICP<br>• $PbtO_2$<br>• TCD<br>• CMD
Veldeman et al., 2020 (109)	aSAH	Assess the impact of invasive neuromonitoring on the detection rate of DCI and its influence on patient outcome.	190	2010 - 2018	• ICP<br>• $PbtO_2$<br>• TCD<br>• CMD

Ref.	Dx.	Aim or objective(s)	No. of patients (N)	Enrollment period	Modalities
Bogossian et al., 2021 (110)	SAH	Assessed the impact of ICP/$PbtO_2$-guided therapy on neurological outcome in SAH patients; a subgroup analysis including only patients receiving therapies driven by neuromonitoring (ICP-guided vs. ICP/$PbtO_2$ -guided); the impact of ICP/ $PbtO_2$ -guided therapy on hospital mortality; subgroup analysis of aneurysmal SAH patients.	163	Jun 2014 - Mar 2020	• ICP<br>• $PbtO_2$
Fergusson et al., 2021 (111)	HIBI	Evaluating whether goal-directed therapy using invasive neuromonitoring is associated with improved neurological outcome in hypoxic ischemic brain injury patients after cardiac arrest.	65	Jul 2016 - Nov 2019	• ICP<br>• Cerebral T<br>• $PbtO_2$<br>• $SvjO_2$<br>• sEEG [a]
Khellaf et al., 2021 (102)	TBI	Assess the feasibility and utility of a systematic protocol to identify, characterize and treat derangements of CMD-derived L/P-ratio>25 within MMM; target a novel focally administered intervention, succinate in patients identified as having mitochondrial derangement based on MMM parameters.	33	Sep 2017 - Jun 2019	• ICP<br>• $PbtO_2$<br>• CMD
Winberg et al., 2022 (112)	aSAH	Evaluate the use and clinical efficacy of CMD-based interventions in the NICU of patients with SAH and to assess the prevalence of DCI-related infarction.	49	Jan 2018 - Dec 2020	• ICP<br>• CMD<br>• TCD

[a] Other modalities. Modalities described in the study but not part of the study aim/objective(s). These modalities were either used for clinical management or not part of our included modalities, but continuous or daily updated monitor.

ABP = arterial blood pressure; AIS = acute ischemic stroke; CA = cerebral autoregulation; Cerebral T = cerebral temperature; CIAN = Continuous intra-arterial nimodipine; CMD = cerebral microdialysis; CPP = cerebral perfusion pressure; CT = computer tomography; DCI = diffuse cerebral ischemia; dEEG = depth electroencephalography; ECoG = electrocorticography; FiO_2 = fraction of inspired oxygen; HIBI: hypoxic ischemic brain injury following cardiac arrest; ICH = intracerebral hemorrhage; ICP = intracranial pressure; ICU = intensive care unit; L/P-ratio = lactate/pyruvate ratio; MRI = magnetic Resonance Imaging; NICU = neuroscience intensive care unit; NIRS = near-infrared spectroscopy; No. = number; PaO_2 = partial arterial oxygenation pressure; PaO_2 = partial pressure of oxygen; $PbtO_2$ = partial pressure of brain tissue oxygenation; PRx = pressure reactivity index; rCBF = regional cerebral blood flow; (a)SAH = (aneurysmal) subarachnoid hemorrhage; sEEG = surface electroencephalography; $SvjO_2$ = jugular bulb venous oximetry; TBI = traumatic brain injury; TCD = transcranial Doppler.

REFERENCES

1. Bouzat P, Marques-Vidal P, Zerlauth J-B, Sala N, Suys T, Schoettker P, et al. Accuracy of brain multimodal monitoring to detect cerebral hypoperfusion after traumatic brain injury*. Crit Care Med. 2015 Feb;43(2):445–52.

2. Esnault P, Boret H, Montcriol A, Carre E, Prunet B, Bordes J, et al. Assessment of cerebral oxygenation in neurocritical care patients: comparison of a new four wavelengths forehead regional saturation in oxygen sensor (EQUANOX®) with brain tissue oxygenation. A prospective observational study. Minerva Anestesiol. 2015 Aug;81(8):876–84.

3. Helbok R, Schiefecker AJ, Beer R, Dietmann A, Antunes AP, Sohm F, et al. Early brain injury after aneurysmal subarachnoid hemorrhage: a multimodal neuromonitoring study. Crit Care. 2015 Mar;19(1):75.

4. Tackla R, Hinzman JM, Foreman B, Magner M, Andaluz N, Hartings JA. Assessment of Cerebrovascular Autoregulation Using Regional Cerebral Blood Flow in Surgically Managed Brain Trauma Patients. Neurocrit Care. 2015 Dec;23(3):339–46.

5. Tholance Y, Barcelos GK, Dailler F, Renaud B, Marinesco S, Perret-Liaudet A. Biochemical neuromonitoring of poor-grade aneurysmal subarachnoid hemorrhage: comparative analysis of metabolic events detected by cerebral microdialysis and by retrograde jugular vein catheterization. Neurol Res. 2015 Jul;37(7):578–87.

6. Budohoski KP, Czosnyka M, Smielewski P, Varsos G V, Kasprowicz M, Brady KM, et al. Monitoring Cerebral Autoregulation After Subarachnoid Hemorrhage. Acta Neurochir Suppl. 2016;122:199–203.

7. Dias C, Maia I, Cerejo A, Smielewski P, Paiva J-A, Czosnyka M. Plateau Waves of Intracranial Pressure and Multimodal Brain Monitoring. Acta Neurochir Suppl. 2016;122:143–6.

8. Helbok R, Schiefecker AJ, Friberg C, Beer R, Kofler M, Rhomberg P, et al. Spreading depolarizations in patients with spontaneous intracerebral hemorrhage: Association with perihematomal edema progression. J Cereb blood flow Metab Off J Int Soc Cereb Blood Flow Metab. 2017 May;37(5):1871–82.

9. Hifumi T, Kawakita K, Yoda T, Okazaki T, Kuroda Y. Association of brain metabolites with blood lactate and glucose levels with respect to neurological outcomes after out-of-hospital cardiac arrest: A preliminary microdialysis study. Resuscitation. 2017 Jan;110:26–31.

10. Hinzman JM, Wilson JA, Mazzeo AT, Bullock MR, Hartings JA. Excitotoxicity and Metabolic Crisis Are Associated with Spreading Depolarizations in Severe Traumatic Brain Injury Patients. J Neurotrauma. 2016 Oct;33(19):1775–83.

11. Myers RB, Lazaridis C, Jermaine CM, Robertson CS, Rusin CG. Predicting Intracranial

Pressure and Brain Tissue Oxygen Crises in Patients With Severe Traumatic Brain Injury. Crit Care Med. 2016 Sep;44(9):1754–61.

12. Papadopoulos D, Filippidis A, Krommidas G, Vretzakis G, Paterakis K, Komnos A, et al. Regional cerebral blood flow and cellular environment in subarachnoid hemorrhage: A thermal doppler flowmetry and microdialysis study. Neurol Neurochir Pol. 2017;51(1):66–71.

13. Patet C, Quintard H, Zerlauth J-B, Maibach T, Carteron L, Suys T, et al. Bedside cerebral microdialysis monitoring of delayed cerebral hypoperfusion in comatose patients with poor grade aneurysmal subarachnoid haemorrhage. J Neurol Neurosurg Psychiatry. 2017 Apr;88(4):332–8.

14. Vanga SR, Korlakunta H, Duthuluru S, Bommana S, Narotam P, Ryschon K, et al. Partial Brain Tissue Oxygen Levels Predict Arrhythmia and Prognosis in Patients With Brain Injury. Am J Ther. 2016;23(6):e1781–7.

15. Vespa P, Tubi M, Claassen J, Buitrago-Blanco M, McArthur D, Velazquez AG, et al. Metabolic crisis occurs with seizures and periodic discharges after brain trauma. Ann Neurol. 2016 Apr;79(4):579–90.

16. Winkler MK, Dengler N, Hecht N, Hartings JA, Kang EJ, Major S, et al. Oxygen availability and spreading depolarizations provide complementary prognostic information in neuromonitoring of aneurysmal subarachnoid hemorrhage patients. J Cereb blood flow Metab Off J Int Soc Cereb Blood Flow Metab. 2017 May;37(5):1841–56.

17. Carteron L, Patet C, Solari D, Messerer M, Daniel RT, Eckert P, et al. Non-Ischemic Cerebral Energy Dysfunction at the Early Brain Injury Phase following Aneurysmal Subarachnoid Hemorrhage. Front Neurol. 2017;8:325.

18. Foreman B, Albers D, Schmidt JM, Falo CM, Velasquez A, Connolly ES, et al. Intracortical electrophysiological correlates of blood flow after severe SAH: A multimodality monitoring study. J Cereb blood flow Metab Off J Int Soc Cereb Blood Flow Metab. 2018 Mar;38(3):506–17.

19. Nyholm L, Howells T, Lewén A, Hillered L, Enblad P. The influence of hyperthermia on intracranial pressure, cerebral oximetry and cerebral metabolism in traumatic brain injury. Ups J Med Sci. 2017 Aug;122(3):177–84.

20. Pinczolits A, Zdunczyk A, Dengler NF, Hecht N, Kowoll CM, Dohmen C, et al. Standard-sampling microdialysis and spreading depolarizations in patients with malignant hemispheric stroke. J Cereb blood flow Metab Off J Int Soc Cereb Blood Flow Metab. 2017 May;37(5):1896–905.

21. Schiefecker AJ, Kofler M, Gaasch M, Beer R, Unterberger I, Pfausler B, et al. Brain temperature but not core temperature increases during spreading depolarizations in patients with spontaneous intracerebral hemorrhage. J Cereb blood flow Metab Off J Int Soc Cereb Blood Flow Metab. 2018 Mar;38(3):549–58.

22. Witsch J, Frey H-P, Schmidt JM, Velazquez A, Falo CM, Reznik M, et al.

Electroencephalographic Periodic Discharges and Frequency-Dependent Brain Tissue Hypoxia in Acute Brain Injury. JAMA Neurol. 2017 Mar;74(3):301–9.

23. Chen Y, Xu W, Wang L, Yin X, Cao J, Deng F, et al. Transcranial Doppler combined with quantitative EEG brain function monitoring and outcome prediction in patients with severe acute intracerebral hemorrhage. Crit Care. 2018 Feb;22(1):36.

24. Dellazizzo L, Demers S-P, Charbonney E, Williams V, Serri K, Albert M, et al. Minimal PaO2 threshold after traumatic brain injury and clinical utility of a novel brain oxygenation ratio. J Neurosurg. 2018 Nov;1–9.

25. Dreier JP, Major S, Foreman B, Winkler MKL, Kang E-J, Milakara D, et al. Terminal spreading depolarization and electrical silence in death of human cerebral cortex. Ann Neurol. 2018 Feb;83(2):295–310.

26. Foreman B, Ngwenya LB, Stoddard E, Hinzman JM, Andaluz N, Hartings JA. Safety and Reliability of Bedside, Single Burr Hole Technique for Intracranial Multimodality Monitoring in Severe Traumatic Brain Injury. Neurocrit Care. 2018 Dec;29(3):469–80.

27. Lückl J, Lemale CL, Kola V, Horst V, Khojasteh U, Oliveira-Ferreira AI, et al. The negative ultraslow potential, electrophysiological correlate of infarction in the human cortex. Brain. 2018 Jun;141(6):1734–52.

28. Morris NA, Robinson D, Schmidt JM, Frey HP, Park S, Agarwal S, et al. Hunt-Hess 5 subarachnoid haemorrhage presenting with cardiac arrest is associated with larger volume bleeds. Resuscitation. 2018 Feb;123:71–6.

29. Alkhachroum A, Megjhani M, Terilli K, Rubinos C, Ford J, Wallace BK, et al. Hyperemia in subarachnoid hemorrhage patients is associated with an increased risk of seizures. J Cereb blood flow Metab Off J Int Soc Cereb Blood Flow Metab. 2020 Jun;40(6):1290–9.

30. Bailey RL, Quattrone F, Curtin C, Frangos S, Maloney-Wilensky E, Levine JM, et al. The Safety of Multimodality Monitoring Using a Triple-Lumen Bolt in Severe Acute Brain Injury. World Neurosurg. 2019 Oct;130:e62–7.

31. Calviello LA, Zeiler FA, Donnelly J, Uryga A, de Riva N, Smielewski P, et al. Estimation of pulsatile cerebral arterial blood volume based on transcranial doppler signals. Med Eng Phys. 2019 Dec;74:23–32.

32. Davie S, Mutch WAC, Monterola M, Fidler K, Funk DJ. The Incidence and Magnitude of Cerebral Desaturation in Traumatic Brain Injury: An Observational Cohort Study. J Neurosurg Anesthesiol. 2021 Jul;33(3):258–62.

33. Donnelly J, Smielewski P, Adams H, Zeiler FA, Cardim D, Liu X, et al. Observations on the Cerebral Effects of Refractory Intracranial Hypertension After Severe Traumatic Brain Injury. Neurocrit Care. 2020 Apr;32(2):437–47.

34. Forsse A, Nielsen TH, Mølstrøm S, Hjelmborg J, Nielsen KS, Nygaard KH, et al. A Prospective Observational Feasibility Study of Jugular Bulb Microdialysis in Subarachnoid

Hemorrhage. Neurocrit Care. 2020 Aug;33(1):241–55.

35. Launey Y, Fryer TD, Hong YT, Steiner LA, Nortje J, Veenith T V, et al. Spatial and Temporal Pattern of Ischemia and Abnormal Vascular Function Following Traumatic Brain Injury. JAMA Neurol. 2020 Mar;77(3):339–49.

36. Rajagopalan S, Cruz Navarro J, Baghshomali S, Kirschen M, Greer D, Kofke WA, et al. Physiological Signatures of Brain Death Uncovered by Intracranial Multimodal Neuromonitoring. J Neurosurg Anesthesiol. 2021 Oct;33(4):347–50.

37. Silverman A, Kodali S, Strander S, Gilmore EJ, Kimmel A, Wang A, et al. Deviation From Personalized Blood Pressure Targets Is Associated With Worse Outcome After Subarachnoid Hemorrhage. Stroke. 2019 Oct;50(10):2729–37.

38. Svedung Wettervik T, Howells T, Ronne-Engström E, Hillered L, Lewén A, Enblad P, et al. High Arterial Glucose is Associated with Poor Pressure Autoregulation, High Cerebral Lactate/Pyruvate Ratio and Poor Outcome Following Traumatic Brain Injury. Neurocrit Care. 2019 Dec;31(3):526–33.

39. Addis A, Gaasch M, Schiefecker AJ, Kofler M, Ianosi B, Rass V, et al. Brain temperature regulation in poor-grade subarachnoid hemorrhage patients - A multimodal neuromonitoring study. J Cereb blood flow Metab Off J Int Soc Cereb Blood Flow Metab. 2021 Feb;41(2):359–68.

40. Gagnon A, Laroche M, Williamson D, Giroux M, Giguère J-F, Bernard F. Incidence and characteristics of cerebral hypoxia after craniectomy in brain-injured patients: a cohort study. J Neurosurg. 2020 Nov;1–8.

41. Marini CP, Stoller C, McNelis J, Del Deo V, Prabhakaran K, Petrone P. Correlation of brain flow variables and metabolic crisis: a prospective study in patients with severe traumatic brain injury. Eur J trauma Emerg Surg Off Publ Eur Trauma Soc. 2020 Jul;

42. Rass V, Huber L, Ianosi B-A, Kofler M, Lindner A, Picetti E, et al. The Effect of Temperature Increases on Brain Tissue Oxygen Tension in Patients with Traumatic Brain Injury: A Collaborative European NeuroTrauma Effectiveness Research in Traumatic Brain Injury Substudy. Ther Hypothermia Temp Manag. 2021 Jun;11(2):122–31.

43. Robba C, Asgari S, Gupta A, Badenes R, Sekhon M, Bequiri E, et al. Lung Injury Is a Predictor of Cerebral Hypoxia and Mortality in Traumatic Brain Injury. Front Neurol. 2020;11:771.

44. Sekhon MS, Ainslie PN, Menon DK, Thiara SS, Cardim D, Gupta AK, et al. Brain Hypoxia Secondary to Diffusion Limitation in Hypoxic Ischemic Brain Injury Postcardiac Arrest. Crit Care Med. 2020 Mar;48(3):378–84.

45. Svedung Wettervik T, Engquist H, Howells T, Rostami E, Hillered L, Enblad P, et al. Arterial lactate in traumatic brain injury - Relation to intracranial pressure dynamics, cerebral energy metabolism and clinical outcome. J Crit Care. 2020 Dec;60:218–25.

46. Zeiler FA, Beqiri E, Cabeleira M, Hutchinson PJ, Stocchetti N, Menon DK, et al. Brain Tissue

Oxygen and Cerebrovascular Reactivity in Traumatic Brain Injury: A Collaborative European NeuroTrauma Effectiveness Research in Traumatic Brain Injury Exploratory Analysis of Insult Burden. J Neurotrauma. 2020 Sep;37(17):1854–63.

47. Zeiler FA, Ercole A, Cabeleira M, Stocchetti N, Hutchinson PJ, Smielewski P, et al. Descriptive analysis of low versus elevated intracranial pressure on cerebral physiology in adult traumatic brain injury: a CENTER-TBI exploratory study. Acta Neurochir (Wien). 2020 Nov;162(11):2695–706.

48. Zeiler FA, Cabeleira M, Hutchinson PJ, Stocchetti N, Czosnyka M, Smielewski P, et al. Evaluation of the relationship between slow-waves of intracranial pressure, mean arterial pressure and brain tissue oxygen in TBI: a CENTER-TBI exploratory analysis. J Clin Monit Comput. 2020 May;

49. Balu R, Rajagopalan S, Baghshomali S, Kirschen M, Amurthur A, Kofke WA, et al. Cerebrovascular pressure reactivity and intracranial pressure are associated with neurologic outcome after hypoxic-ischemic brain injury. Resuscitation. 2021 Jul;164:114–21.

50. Al Barajraji M, Bogossian E, Dewitte O, Gaspard N, El Hadwe S, Minini A, et al. Safety profile of an intracranial multimodal monitoring bolt system for neurocritical care: a single-center experience. Acta Neurochir (Wien). 2021 Dec;163(12):3259–66.

51. Birg T, Ortolano F, Wiegers EJA, Smielewski P, Savchenko Y, Ianosi BA, et al. Brain Temperature Influences Intracranial Pressure and Cerebral Perfusion Pressure After Traumatic Brain Injury: A CENTER-TBI Study. Neurocrit Care. 2021 Dec;35(3):651–61.

52. Guilfoyle MR, Helmy A, Donnelly J, Stovell MG, Timofeev I, Pickard JD, et al. Characterising the dynamics of cerebral metabolic dysfunction following traumatic brain injury: A microdialysis study in 619 patients. PLoS One. 2021;16(12):e0260291.

53. Hoiland RL, Ainslie PN, Wellington CL, Cooper J, Stukas S, Thiara S, et al. Brain Hypoxia Is Associated With Neuroglial Injury in Humans Post-Cardiac Arrest. Circ Res. 2021 Aug;129(5):583–97.

54. Kieninger M, Meichelböck K, Bele S, Bründl E, Graf B, Schmidt NO, et al. Brain multimodality monitoring in patients suffering from acute aneurysmal subarachnoid hemorrhage: clinical value and complications. J Integr Neurosci. 2021 Sep;20(3):703–10.

55. Kuo L-T, Lu H-Y, Huang AP-H. Prognostic Value of Circadian Rhythm of Brain Temperature in Traumatic Brain Injury. J Pers Med. 2021 Jun;11(7).

56. Lindner A, Rass V, Ianosi B-A, Schiefecker AJ, Kofler M, Gaasch M, et al. Individualized blood pressure targets in the postoperative care of patients with intracerebral hemorrhage. J Neurosurg. 2021 Apr;1–10.

57. Mueller TM, Gollwitzer S, Hopfengärtner R, Rampp S, Lang JD, Stritzelberger J, et al. Alpha power decrease in quantitative EEG detects development of cerebral infarction after subarachnoid hemorrhage early. Clin Neurophysiol Off J Int Fed Clin Neurophysiol. 2021 Mar;

58. Qi Y, Xing Y, Wang L, Zhang J, Cao Y, Liu L, et al. Multimodal Monitoring in Large Hemispheric Infarction: Quantitative Electroencephalography Combined With Transcranial Doppler for Prognosis Prediction. Front Neurol. 2021;12:724571.

59. Rajagopalan S, Baker W, Mahanna-Gabrielli E, Kofke AW, Balu R. Hierarchical Cluster Analysis Identifies Distinct Physiological States After Acute Brain Injury. Neurocrit Care. 2022 Apr;36(2):630–9.

60. Schumm L, Lemale CL, Major S, Hecht N, Nieminen-Kelhä M, Zdunczyk A, et al. Physiological variables in association with spreading depolarizations in the late phase of ischemic stroke. J Cereb blood flow Metab Off J Int Soc Cereb Blood Flow Metab. 2022 Jan;42(1):121–35.

61. Wettervik TS, Howells T, Hillered L, Rostami E, Lewén A, Enblad P. Autoregulatory or Fixed Cerebral Perfusion Pressure Targets in Traumatic Brain Injury: Determining Which Is Better in an Energy Metabolic Perspective. J Neurotrauma. 2021 Mar;

62. Wettervik TS, Engquist H, Howells T, Lenell S, Rostami E, Hillered L, et al. Arterial Oxygenation in Traumatic Brain Injury-Relation to Cerebral Energy Metabolism, Autoregulation, and Clinical Outcome. J Intensive Care Med. 2021 Sep;36(9):1075–83.

63. Svedung Wettervik TM, Engquist H, Lenell S, Howells T, Hillered L, Rostami E, et al. Systemic Hyperthermia in Traumatic Brain Injury-Relation to Intracranial Pressure Dynamics, Cerebral Energy Metabolism, and Clinical Outcome. J Neurosurg Anesthesiol. 2021 Oct;33(4):329–36.

64. Dreier JP, Winkler MKL, Major S, Horst V, Lublinsky S, Kola V, et al. Spreading depolarizations in ischaemia after subarachnoid haemorrhage, a diagnostic phase III study. Brain. 2022 May;145(4):1264–84.

65. Owen B, Vangala A, Fritch C, Alsarah AA, Jones T, Davis H, et al. Cerebral Autoregulation Correlation With Outcomes and Spreading Depolarization in Aneurysmal Subarachnoid Hemorrhage. Stroke. 2022 Jun;53(6):1975–83.

66. Svedung Wettervik T, Hånell A, Howells T, Ronne-Engström E, Enblad P, Lewén A. Association of Arterial Metabolic Content with Cerebral Blood Flow Regulation and Cerebral Energy Metabolism-A Multimodality Analysis in Aneurysmal Subarachnoid Hemorrhage. J Intensive Care Med. 2022 Feb;8850666221080054.

67. Svedung Wettervik T, Hånell A, Howells T, Ronne-Engström E, Lewén A, Enblad P. Intracranial pressure- and cerebral perfusion pressure threshold-insults in relation to cerebral energy metabolism in aneurysmal subarachnoid hemorrhage. Acta Neurochir (Wien). 2022 Apr;164(4):1001–14.

68. Yang B, Liang X, Wu Z, Sun X, Shi Q, Zhan Y, et al. APOE gene polymorphism alters cerebral oxygen saturation and quantitative EEG in early-stage traumatic brain injury. Clin Neurophysiol Off J Int Fed Clin Neurophysiol. 2022 Apr;136:182–90.

69. Flynn LMC, Rhodes J, Andrews PJD. Therapeutic Hypothermia Reduces Intracranial Pressure

and Partial Brain Oxygen Tension in Patients with Severe Traumatic Brain Injury: Preliminary Data from the Eurotherm3235 Trial. Ther Hypothermia Temp Manag. 2015 Sep;5(3):143–51.

70. Kurtz P, Helbok R, Claassen J, Schmidt JM, Fernandez L, Stuart RM, et al. The Effect of Packed Red Blood Cell Transfusion on Cerebral Oxygenation and Metabolism After Subarachnoid Hemorrhage. Neurocrit Care. 2016 Feb;24(1):118–21.

71. Sekhon MS, Griesdale DE, Czosnyka M, Donnelly J, Liu X, Aries MJ, et al. The Effect of Red Blood Cell Transfusion on Cerebral Autoregulation in Patients with Severe Traumatic Brain Injury. Neurocrit Care. 2015 Oct;23(2):210–6.

72. Ghosh A, Highton D, Kolyva C, Tachtsidis I, Elwell CE, Smith M. Hyperoxia results in increased aerobic metabolism following acute brain injury. J Cereb blood flow Metab Off J Int Soc Cereb Blood Flow Metab. 2017 Aug;37(8):2910–20.

73. McCredie VA, Piva S, Santos M, Xiong W, de Oliveira Manoel AL, Rigamonti A, et al. The Impact of Red Blood Cell Transfusion on Cerebral Tissue Oxygen Saturation in Severe Traumatic Brain Injury. Neurocrit Care. 2017 Apr;26(2):247–55.

74. Sahoo S, Sheshadri V, Sriganesh K, Madhsudana Reddy KR, Radhakrishnan M, Umamaheswara Rao GS. Effect of Hyperoxia on Cerebral Blood Flow Velocity and Regional Oxygen Saturation in Patients Operated on for Severe Traumatic Brain Injury-The Influence of Cerebral Blood Flow Autoregulation. World Neurosurg. 2017 Feb;98:211–6.

75. Westermaier T, Stetter C, Kunze E, Willner N, Holzmeier J, Weiland J, et al. Controlled Hypercapnia Enhances Cerebral Blood Flow and Brain Tissue Oxygenation After Aneurysmal Subarachnoid Hemorrhage: Results of a Phase 1 Study. Neurocrit Care. 2016 Oct;25(2):205–14.

76. Zhang Y, Liu X, Steiner L, Smielewski P, Feen E, Pickard JD, et al. Correlation Between Cerebral Autoregulation and Carbon Dioxide Reactivity in Patients with Traumatic Brain Injury. Acta Neurochir Suppl. 2016;122:205–9.

77. Kofler M, Schiefecker AJ, Beer R, Gaasch M, Rhomberg P, Stover J, et al. Enteral nutrition increases interstitial brain glucose levels in poor-grade subarachnoid hemorrhage patients. J Cereb blood flow Metab Off J Int Soc Cereb Blood Flow Metab. 2018 Mar;38(3):518–27.

78. Carteron L, Solari D, Patet C, Quintard H, Miroz J-P, Bloch J, et al. Hypertonic Lactate to Improve Cerebral Perfusion and Glucose Availability After Acute Brain Injury. Crit Care Med. 2018 Oct;46(10):1649–55.

79. Jakkula P, Pettilä V, Skrifvars MB, Hästbacka J, Loisa P, Tiainen M, et al. Targeting low-normal or high-normal mean arterial pressure after cardiac arrest and resuscitation: a randomised pilot trial. Intensive Care Med. 2018;2091–101.

80. Brandi G, Stocchetti N, Pagnamenta A, Stretti F, Steiger P, Klinzing S. Cerebral metabolism is not affected by moderate hyperventilation in patients with traumatic brain injury. Crit Care. 2019 Feb;23(1):45.

81. Sekhon MS, Gooderham P, Menon DK, Brasher PMA, Foster D, Cardim D, et al. The Burden of Brain Hypoxia and Optimal Mean Arterial Pressure in Patients With Hypoxic Ischemic Brain Injury After Cardiac Arrest. Crit Care Med. 2019 Jul;47(7):960–9.

82. Svedung Wettervik T, Howells T, Hillered L, Nilsson P, Engquist H, Lewén A, et al. Mild Hyperventilation in Traumatic Brain Injury-Relation to Cerebral Energy Metabolism, Pressure Autoregulation, and Clinical Outcome. World Neurosurg. 2020 Jan;133:e567–75.

83. Burnol L, Payen J-F, Francony G, Skaare K, Manet R, Morel J, et al. Impact of Head-of-Bed Posture on Brain Oxygenation in Patients with Acute Brain Injury: A Prospective Cohort Study. Neurocrit Care. 2021 Dec;35(3):662–8.

84. Dagod G, Roustan J-P, Bringuier-Branchereau S, Ridolfo J, Martinez O, Capdevila X, et al. Effect of a temporary lying position on cerebral hemodynamic and cerebral oxygenation parameters in patients with severe brain trauma. Acta Neurochir (Wien). 2021 Sep;163(9):2595–602.

85. Hosmann A, Angelmayr C, Hopf A, Rauscher S, Brugger J, Ritscher L, et al. Detrimental effects of intrahospital transport on cerebral metabolism in patients suffering severe aneurysmal subarachnoid hemorrhage. J Neurosurg. 2021 Mar;1–8.

86. Kovacs M, Peluso L, Njimi H, De Witte O, Gouvêa Bogossian E, Quispe Cornejo A, et al. Optimal Cerebral Perfusion Pressure Guided by Brain Oxygen Pressure Measurement. Front Neurol. 2021;12:732830.

87. Rass V, Bogossian EG, Ianosi B-A, Peluso L, Kofler M, Lindner A, et al. The effect of the volemic and cardiac status on brain oxygenation in patients with subarachnoid hemorrhage: a bi-center cohort study. Ann Intensive Care. 2021 Dec;11(1):176.

88. Stetter C, Weidner F, Lilla N, Weiland J, Kunze E, Ernestus R-I, et al. Therapeutic hypercapnia for prevention of secondary ischemia after severe subarachnoid hemorrhage: physiological responses to continuous hypercapnia. Sci Rep. 2021 Jun;11(1):11715.

89. Bernini A, Miroz J-P, Abed-Maillard S, Favre E, Iaquaniello C, Ben-Hamouda N, et al. Hypertonic lactate for the treatment of intracranial hypertension in patients with acute brain injury. Sci Rep. 2022 Feb;12(1):3035.

90. Gouvêa Bogossian E, Rass V, Lindner A, Iaquaniello C, Miroz JP, Cavalcante Dos Santos E, et al. Factors Associated With Brain Tissue Oxygenation Changes After RBC Transfusion in Acute Brain Injury Patients. Crit Care Med. 2022 Jun;50(6):e539–47.

91. Gargadennec T, Ferraro G, Chapusette R, Chapalain X, Bogossian E, Van Wettere M, et al. Detection of cerebral hypoperfusion with a dynamic hyperoxia test using brain oxygenation pressure monitoring. Crit Care. 2022 Feb;26(1):35.

92. Hosmann A, Schnackenburg P, Rauscher S, Hopf A, Bohl I, Engel A, et al. Brain Tissue Oxygen Response as Indicator for Cerebral Lactate Levels in Aneurysmal Subarachnoid Hemorrhage Patients. J Neurosurg Anesthesiol. 2022 Apr;34(2):193–200.

93. Hockel K, Diedler J, Steiner J, Birkenhauer U, Danz S, Ernemann U, et al. Long-Term,

Continuous Intra-Arterial Nimodipine Treatment of Severe Vasospasm After Aneurysmal Subarachnoid Hemorrhage. World Neurosurg. 2016 Apr;88:104–12.

94. Akbik OS, Krasberg M, Nemoto EM, Yonas H. Effect of Cerebrospinal Fluid Drainage on Brain Tissue Oxygenation in Traumatic Brain Injury. J Neurotrauma. 2017 Nov;34(22):3153–7.

95. Albanna W, Weiss M, Müller M, Brockmann MA, Rieg A, Conzen C, et al. Endovascular Rescue Therapies for Refractory Vasospasm After Subarachnoid Hemorrhage: A Prospective Evaluation Study Using Multimodal, Continuous Event Neuromonitoring. Neurosurgery. 2017 Jun;80(6):942–9.

96. Hockel K, Diedler J, Steiner J, Birkenhauer U, Ernemann U, Schuhmann MU. Effect of Intra-Arterial and Intravenous Nimodipine Therapy of Cerebral Vasospasm After Subarachnoid Hemorrhage on Cerebrovascular Reactivity and Oxygenation. World Neurosurg. 2017 May;101:372–8.

97. Lubillo ST, Parrilla DM, Blanco J, Morera J, Dominguez J, Belmonte F, et al. Prognostic value of changes in brain tissue oxygen pressure before and after decompressive craniectomy following severe traumatic brain injury. J Neurosurg. 2018 May;128(5):1538–46.

98. Ding C-Y, Kang D-Z, Wang Z-L, Lin Y-X, Jiang C-Z, Yu L-H, et al. Serum Ngb (Neuroglobin) Is Associated With Brain Metabolism and Functional Outcome of Aneurysmal Subarachnoid Hemorrhage. Stroke. 2019 Jul;50(7):1887–90.

99. Hosmann A, Wang W-T, Dodier P, Bavinzski G, Engel A, Herta J, et al. The Impact of Intra-Arterial Papaverine-Hydrochloride on Cerebral Metabolism and Oxygenation for Treatment of Delayed-Onset Post-Subarachnoid Hemorrhage Vasospasm. Neurosurgery. 2020 Sep;87(4):712–9.

100. Ianosi B, Rass V, Gaasch M, Huber L, Lindner A, Hackl WO, et al. An Observational Study on the Use of Intravenous Non-Opioid Analgesics and Antipyretics in Poor-Grade Subarachnoid Hemorrhage: Effects on Hemodynamics and Systemic and Brain Temperature. Ther Hypothermia Temp Manag. 2020 Mar;10(1):27–36.

101. Koskinen L-OD, Sundström N, Hägglund L, Eklund A, Olivecrona M. Prostacyclin Affects the Relation Between Brain Interstitial Glycerol and Cerebrovascular Pressure Reactivity in Severe Traumatic Brain Injury. Neurocrit Care. 2019 Dec;31(3):494–500.

102. Khellaf A, Garcia NM, Tajsic T, Alam A, Stovell MG, Killen MJ, et al. Focally administered succinate improves cerebral metabolism in traumatic brain injury patients with mitochondrial dysfunction. J Cereb blood flow Metab Off J Int Soc Cereb Blood Flow Metab. 2022 Jan;42(1):39–55.

103. Bele S, Proescholdt MA, Hochreiter A, Schuierer G, Scheitzach J, Wendl C, et al. Continuous intra-arterial nimodipine infusion in patients with severe refractory cerebral vasospasm after aneurysmal subarachnoid hemorrhage: a feasibility study and outcome results. Acta Neurochir (Wien). 2015 Dec;157(12):2041–50.

104. Lin C-M, Lin M-C, Huang S-J, Chang C-K, Chao D-P, Lui T-N, et al. A Prospective Randomized Study of Brain Tissue Oxygen Pressure-Guided Management in Moderate and Severe Traumatic Brain Injury Patients. Biomed Res Int. 2015;2015:529580.

105. Okonkwo DO, Shutter LA, Moore C, Temkin NR, Puccio AM, Madden CJ, et al. Brain Oxygen Optimization in Severe Traumatic Brain Injury Phase-II: A Phase II Randomized Trial*. 2017;1907–14.

106. Rass V, Solari D, Ianosi B, Gaasch M, Kofler M, Schiefecker AJ, et al. Protocolized Brain Oxygen Optimization in Subarachnoid Hemorrhage. Neurocrit Care. 2019 Oct;31(2):263–72.

107. Sekhon MS, Gooderham P, Toyota B, Kherzi N, Hu V, Dhingra VK, et al. Implementation of Neurocritical Care Is Associated With Improved Outcomes in Traumatic Brain Injury. Can J Neurol Sci Le J Can des Sci Neurol. 2017 Jul;44(4):350–7.

108. Veldeman M, Albanna W, Weiss M, Conzen C, Schmidt TP, Clusmann H, et al. Treatment of Delayed Cerebral Ischemia in Good-Grade Subarachnoid Hemorrhage: Any Role for Invasive Neuromonitoring? Neurocrit Care. 2020 Dec;

109. Veldeman M, Albanna W, Weiss M, Conzen C, Schmidt TP, Schulze-Steinen H, et al. Invasive neuromonitoring with an extended definition of delayed cerebral ischemia is associated with improved outcome after poor-grade subarachnoid hemorrhage. J Neurosurg. 2020 May;134(5):1527–34.

110. Gouvea Bogossian E, Diaferia D, Ndieugnou Djangang N, Menozzi M, Vincent J-L, Talamonti M, et al. Brain tissue oxygenation guided therapy and outcome in non-traumatic subarachnoid hemorrhage. Sci Rep. 2021 Aug;11(1):16235.

111. Fergusson NA, Hoiland RL, Thiara S, Foster D, Gooderham P, Rikhraj K, et al. Goal-Directed Care Using Invasive Neuromonitoring Versus Standard of Care After Cardiac Arrest: A Matched Cohort Study. Crit Care Med. 2021 Aug;49(8):1333–46.

112. Winberg J, Holm I, Cederberg D, Rundgren M, Kronvall E, Marklund N. Cerebral Microdialysis-Based Interventions Targeting Delayed Cerebral Ischemia Following Aneurysmal Subarachnoid Hemorrhage. Neurocrit Care. 2022 Aug;37(1):255–66.
