# Supplementary material for: Cerebral multimodality monitoring in adult neurocritical care patients with acute brain injury: A narrative review
Source: Front Physiol. 2022 Dec 1;13:1071161. doi: 10.3389/fphys.2022.1071161 (PMC9751622; doi:10.3389/fphys.2022.1071161)
Supplement: Supplementary file 2 [file DataSheet1.pdf]

![frontiers]

Supplementary Material

Jeanette Tas, Marek Czosnyka, Iwan C.C. van der Horst et al. Continuous cerebral multimodality monitoring in adult neurocritical care patients with acute brain injury: a narrative review

Supplementary Table S1 | PubMed search terms

Supplementary Table S2 | Definitions for classification of cerebral multimodality monitoring studies

Supplementary Table S3 | Cerebral monitoring modalities of superiority/ inferiority studies

Supplementary Figure S1 | Flowchart study search and identification

Supplementary Figure S2 | Number of cerebral multimodality monitoring studies per year

Supplementary Figure S3 | Number of cerebral monitoring modalities

Supplementary Table S4a | Monitoring setting of 112 cerebral multimodality monitoring studies

Supplementary Table S4b | Study setting and clinical characteristics of 112 cerebral multimodality monitoring studies

Supplementary Table S4c | Study classification of 112 cerebral multimodality monitoring studies

Supplementary Table S1 | PubMed search terms. Each modality includes a query. MeSH Terms were included when available.

ICP	(("intracranial pressure" [MeSH Terms]) OR intracranial pressure OR ICP OR (("cerebral perfusion pressure" [MeSH Terms]) OR "cerebral perfusion pressure" OR CPP)
$PbtO_2$	Partial brain tissue oxygen OR PbtO2
Cerebral T	Cerebral temperature" OR "Brain temperature" OR "cerebral T" OR “Temperature
rCBF	(“Regional cerebral blood flow” OR Regional cerebral blood flow [MeSH Terms]) OR rCBF)
TCD	(Transcranial Doppler [MeSH Terms]) OR Transcranial Doppler OR TCD
$SvjO_2$	Jugular bulb venous oximetry OR Sjvo2 OR SvjO2
CMD	(microdialysis [MeSH Terms]) OR Cerebral microdialysis OR CMD
NIRS	(Near infrared spectroscopy [MeSH Terms]) OR Near infrared spectroscopy OR NIRS
sEEG	(Surface Electroencephalography [MeSH Terms]) OR Surface Electroencephalography; OR EEG OR qEEG
EcOG	(Electrocorticography [MeSH Terms]) OR Electrocorticography; EcOG
dEEG	Depth Electroencephalography OR dEEG

Cerebral T = cerebral temperature; CMD = cerebral microdialysis; dEEG = depth electroencephalography; ECoG = electrocorticography; ICP = intracranial pressure; NIRS = near-infrared spectroscopy; $PbtO_2$ = partial pressure of brain tissue oxygenation; rCBF = regional cerebral blood flow; sEEG = surface electroencephalography; $SvjO_2$ = jugular bulb venous oximetry; TCD = transcranial Doppler

Supplementary Table S2 | Definitions for classification of cerebral multimodal monitoring studies

**Parameter**	**Description**
MMM study	The aim or objective(s) were related to MMM. MMM is defined as the application and reporting of results of at least two modalities without aiming for superiority/inferiority between modalities.<br>Note. An included modality could also be a covariate in a multivariable model or a modality used for the detection of a secondary injury that was primarily studied (e.g., TCD for detection of vasospasm).
Unimodal modalities	The applied modalities for studying the aim/objective(s). TCD also includes transcranial color doppler sonography (TCCD). CBF includes only invasive regional CBF monitoring. NIRS only includes non-invasive monitoring.
Other neuromonitoring modalities	Modalities described in the study but not part of the study aim or objective(s). These modalities were either used for clinical management (e.g., ICP monitoring not part of the study aim/objective(s)) or not part of our included modalities, but continuously or daily updated monitoring (e.g., a non-invasive CBF monitor).
Duration monitoring for data analysis	The period (hr) used for data analysis as described in the studies. This means that the recording time may differ from the analysis period for specific interventions. For example, when only the pre- and post-periods are used for data analysis.
ABP zeroing	The location where the ABP transducer was zeroed when ICP monitoring was available.
Multicentre study	More than one study site included patients for data collection. For example, the Collaborative European Neuro Trauma Effectiveness Research in TBI (CENTER-TBI) and Co-Operative Studies on Brain Injury Depolarizations (COSBID) cohorts.
Study enrollment period	The duration of the study as reported by the authors.
Sample size	The sample size is reported per study, albeit some studies included a combination of diseases. Therefore, studies can appear more than once in the results.
Sex	Percentage of the sample with males.
Multiple pre-defined diseases	More than one acute brain injury, as defined in this review, was described in the study design (TBI, SAH, ICH, AIS, HIBI).

Study classification	*Observations.* The study aim or objective(s) of the study is related to studying a secondary injury by using at least a combination of two modalities. Results are reported as, e.g., clinical outcome comparison between groups or changes within patients (with/without the condition).<br><br>*Interventions.* The study aim or objective(s) of the study is related to external manipulation. This can be either a clinical event (e.g., studying the periods with changes in ventilator settings) or a pre-defined intervention protocol.<br><br>*Intervention, systemic*. Interventions applied with a direct systemic effect (like studying the effect of packed cell red blood transfusion).<br><br>*Intervention, cerebral.* Interventions applied with a direct local cerebral effect (like studying the effect of cerebral spinal fluid drainage on MMM signals).<br><br>*Interventions guided by MMM.* The study aims to compare groups applying a pre-defined management protocol guided by MMM results. This subtype includes randomized controlled trials, pre-/post- introduction of a (clinical) management protocol, or the evaluation of an MMM-guided clinical management protocol.<br><br>*Safety.* The study aim or objective(s) is to test a new MMM modality, method, or intervention with a safety endpoint.<br><br>*Clinical outcome.* The study compares the results of monitoring modalities in patient groups with different clinical outcome (either mortality or functional outcome).
Other diseases/healthy controls	Other than our pre-defined diseases or healthy controls were included in the study. These diseases are only reported per study (Supplementary table 5a-d).
ABP = arterial blood pressure; AIS = acute ischemic stroke; CBF = cerebral blood flow; HIBI = hypoxic-ischemic brain injury following cardiac arrest; ICH = intracerebral hemorrhage; ICP = intracranial pressure; MMM = multimodality monitoring; NIRS = near infrared spectroscopy; SAH = subarachnoid hemorrhage; TBI = traumatic brain injury; TCD = transcranial Doppler	

Supplementary Table S3 | Cerebral monitoring modalities of superiority/ inferiority studies

No. of studies (%)	Total 52 studies	TBI[a] 38 studies	SAH[a] 16 studies	ICH[a] 10 studies	AIS[a] 4 studies	HIBI[a] 6 studies
Unimodal modalities						
I. ICP	40 (77)	32 (84)	11(69)	8 (80)	2 (50)	0
II. $PbtO_2$	13 (25)	8 (21)	6 (38)	2 (20)	0	2 (33)
III. Cerebral T	2 (3.8)	1 (2.6)	1 (6.3)	0	0	0
IV. rCBF	4 (7.7)	4 (11)	0	1 (10)	0	1 (17)
V. TCD	33 (63)	26 (68)	9 (56)	8 (80)	3 (75)	3 (50)
VI. $SvjO_2$	1 (1.9)	1 (2.6)	0	1 (10)	1 (25)	0
VII. CMD	4 (7.7)	1 (2.6)	3 (19)	1 (10)	0	1 (17)
VIII. NIRS	8 (15)	7 (18)	2 (13)	3 (30)	0	3 (50)
IX. sEEG	4 (7.7)	3 (7.9)	2 (13)	1 (10)	1 (25)	0
Invasive neural activity						
X. ECoG	2 (3.8)	2 (5.3)	1 (6.3)	0	1 (25)	0
XI. dEEG	1 (1.9)	1 (2.6)	1 (6.3)	0	0	0

The percentages are reported as whole numbers. The percentages may not count to 100% due to rounding.
[a] Multiple diseases: several studies report more than one disease. These studies are represented for each diagnosis.
AIS = acute ischemic stroke; Cerebral T = cerebral temperature; CMD = cerebral microdialysis; dEEG = depth electroencephalography; ECoG = electrocorticography; MMM = multimodality monitoring; HIBI = hypoxic-ischemic brain injury following cardiac arrest; ICH = intracerebral hemorrhage; ICP = intracranial pressure; NIRS = near-infrared spectroscopy; No. = number; $PbtO_2$ = partial pressure of brain tissue oxygenation; rCBF = regional cerebral blood flow; SAH = subarachnoid hemorrhage; sEEG = surface electroencephalography; $SvjO_2$ = jugular bulb venous oximetry; TBI = traumatic brain injury; TCD = transcranial Doppler.

Supplementary Figure S1 | Flowchart study search and identification

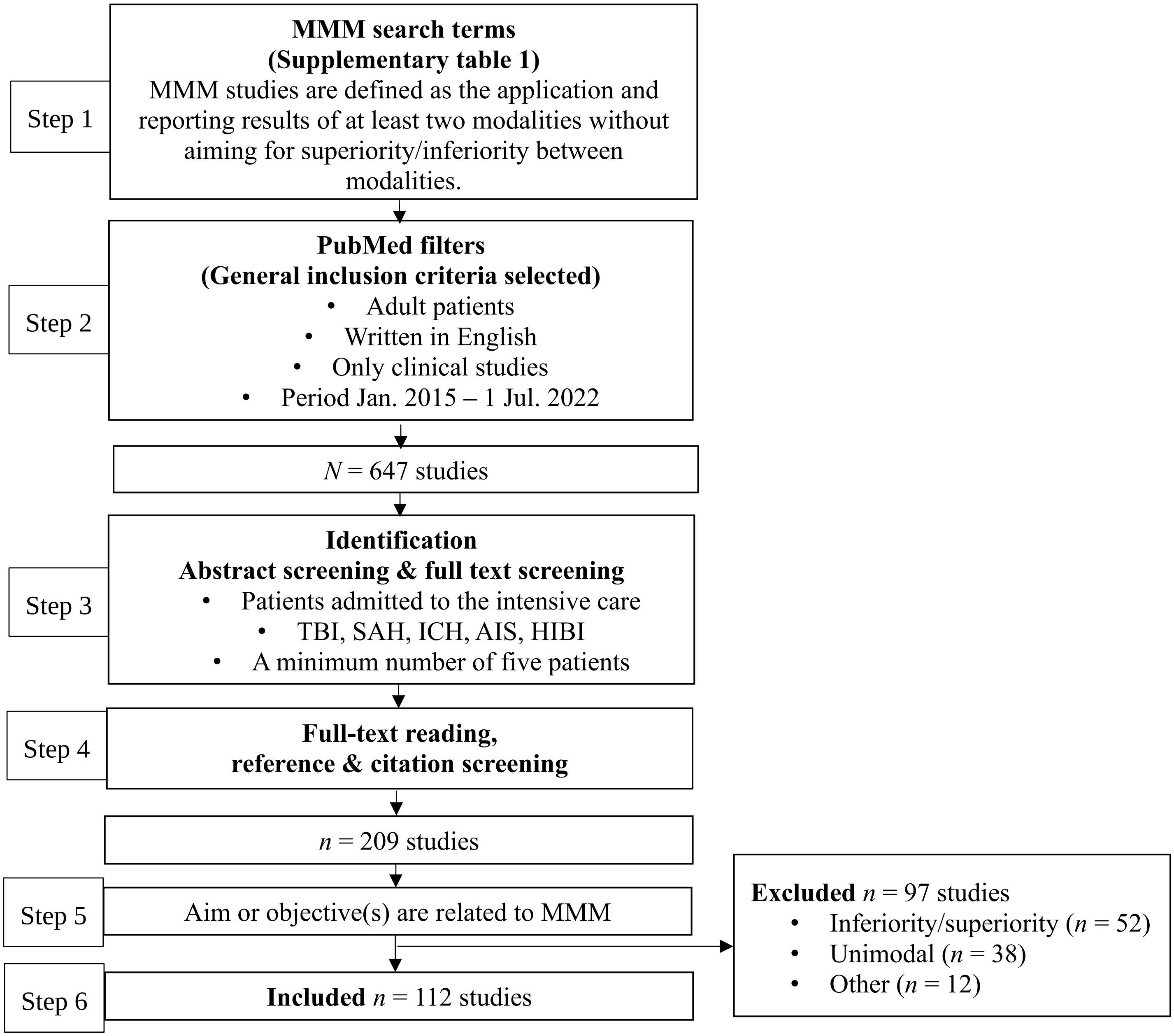

Supplementary Figure S1 legend | Study search and identification flowchart. We used a stepwise approach to select MMM studies described in the methods. The number of excluded studies does not count to 97, as more than one reason could have excluded the study. The excluded group 'unimodal' included studies for which a unimodal aim/objective was described, albeit other modalities were available for, e.g., clinical purposes. Details about the excluded inferiority/superiority studies are given in Supplementary Table S3. The group 'other' included: studies not reporting monitoring results (*n* = 10) (e.g., study protocols); one (exceptional) study was excluded because the authors aimed to evaluate an artifact in ECoG data using $PbtO_2$ monitoring to detect the trigger of the artifact (1), one double publication was excluded (2,3). AIS = acute ischemic stroke; ECoG = electrocorticography; HIBI = hypoxic-ischemic brain injury following cardiac arrest; ICH = intracerebral hemorrhage; MMM = multimodality monitoring; SAH = subarachnoid hemorrhage; $PbtO_2$ = partial pressure of brain tissue oxygenation; TBI = traumatic brain injury.

Supplementary Figure S2 | Number of included cerebral multimodality monitoring studies per year

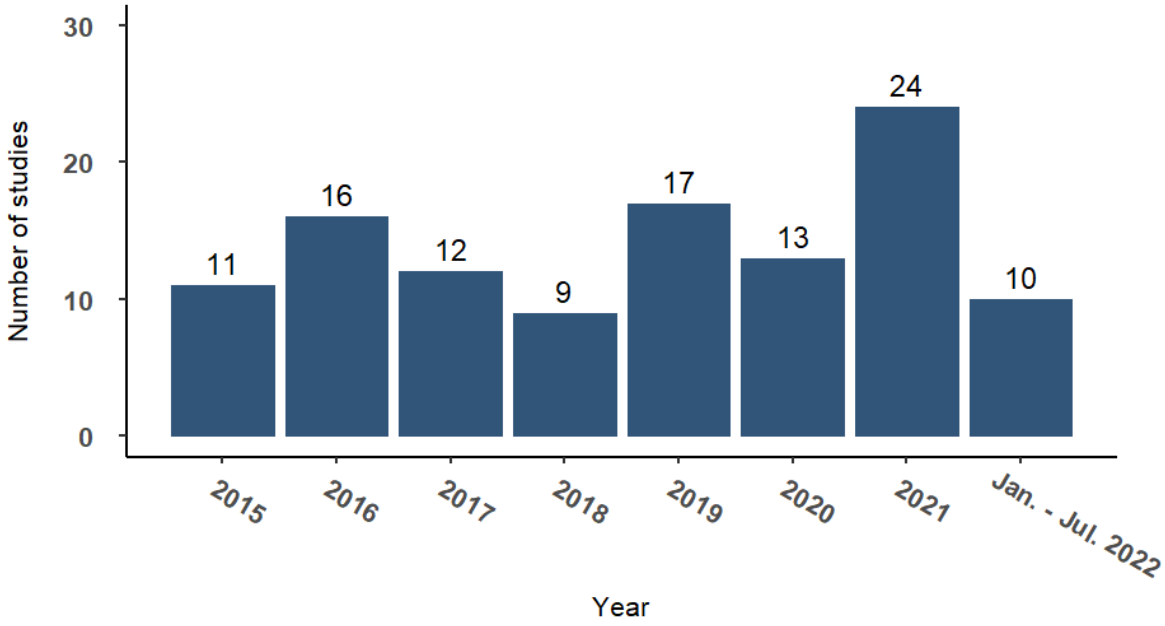

Supplementary Figure S2 legend | The number of cerebral multimodality monitoring studies per year since 2015.

Supplementary Figure | S3 Number of cerebral monitoring modalities

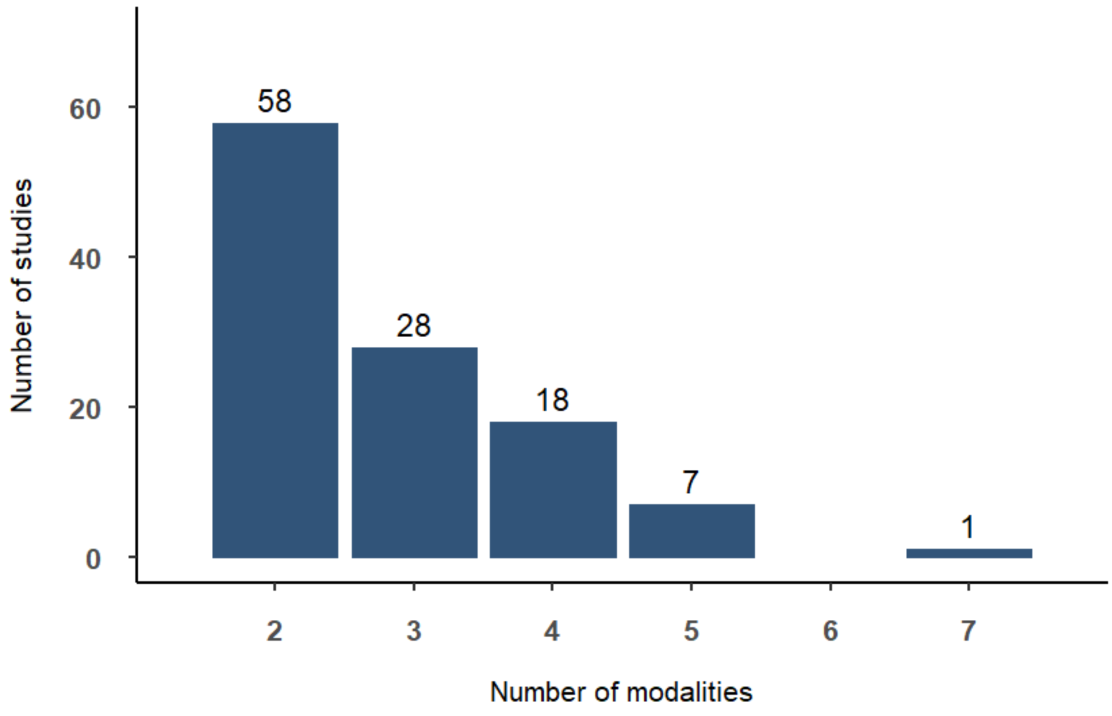

Supplementary Figure S3 legend | The number of cerebral monitoring modalities combined within a study. Each bar represents the number of studies with the corresponding number of combined modalities on the x-axis. On the y-axis, the number of studies is shown. Dreier et al. applied seven modalities, but not in each patient similar combinations (5).

Supplementary Table S4a | Monitoring setting of 112 cerebral multimodality monitoring studies

No. of studies (%)	Total<br>112 studies
Unimodal modalities	
I. ICP	92 (82)
II. $PbtO_2$	71 (63)
III. Cerebral T	16 (14)
IV. rCBF	15 (13)
V. TCD	25 (22)
VI. $SvjO_2$	6 (5.4)
VII. CMD	45 (40)
VIII. NIRS	17 (15)
IX. sEEG	10 (8.9)
Invasive neural activity<br>X. ECoG<br>XI. dEEG	 10 (8.9)<br>7 (6.3)
Other neuromonitoring modalities (not related to the research protocol)	
One modality	29 (26)
Two other modalities	5 (4.5)
Duration monitoring used for data analysis (hr)	
0-1	11 (9.8)
2-12	13 (12)
13-23	7 (6.3)
≥ 24	65 (58)
Not reported	16 (14)
ABP zeroing (when ICP monitoring was applied)	
Heart	13 (12)
Foramen of Monro	9 (8)
Both	1 (1)
Not reported	54 (48)

ABP = arterial blood pressure; Cerebral T = cerebral temperature; CMD = cerebral microdialysis; dEEG = depth electroencephalography; ECoG = electrocorticography; ICP = intracranial pressure; NIRS = near-infrared spectroscopy; No. = number; $PbtO_2$ = partial pressure of brain tissue oxygenation; rCBF = regional cerebral blood flow; sEEG = surface electroencephalography; $SvjO_2$ = jugular bulb venous oximetry; TCD = transcranial Doppler.

Supplementary Table S4b | Study setting and clinical characteristics of 112 cerebral multimodality monitoring studies

No. of studies (%)	Total<br>112 studies
Multicentre studies	22 (20)
Study inclusion period	
0-1 year	23 (21)
2-3 year	26 (23)
4-5 year	13 (12)
≥ 6 year	34 (30)
Not reported	16 (14)
Sample sizes<br>(median, q1 - q3)	36 (20 - 74)
Clinical characteristics	
Sex, male (%), median (q1-q3)	60 (30 - 75)
Age range	
18 - 29 year	1 (0.9)
30 - 39 year	21 (19)
40 - 49 year	29 (26)
50 - 61 year	48 (43)
60 - 69 year	8 (7.1)
Not reported	5 (4.5)
Multiple pre-defined diseases [a]	18 (16)

The percentages are reported as whole numbers. The percentages may not count to 100% due to rounding. Definitions are listed in Supplementary Table S2.
[a] Multiple diseases: some studies report more than one disease. These studies are represented for each diagnosis. Supplementary Table S5a-d lists the studies.
MMM = multimodality monitoring; No. = number; q1-q3 = interquartile range.

Supplementary Table S4c | Study classification of 112 cerebral multimodality monitoring studies

No. of studies (%)	Total<br>112 studies
Study classification	
Observations	68 (61)
Cerebral intervention	10[a] (8.9)
Systemic intervention	24 (18)
Intervention guided by MMM	11[a] (9.8)
Intervention studies - Clinical outcome endpoint	18 (41)
Safety endpoint	17 (15)

[a] One study was classified as both intervention guided by MMM and cerebral intervention (4).
MMM = multimodality monitoring; No. = number

REFERENCES

1. Major S, Gajovic-Eichelmann N, Woitzik J, Dreier JP. Oxygen-Induced and pH-Induced Direct Current Artifacts on Invasive Platinum/Iridium Electrodes for Electrocorticography. Neurocrit Care. 2021 Oct;35(Suppl 2):146–59.

2. Sueiras M, Thonon V, Santamarina E, Sánchez-Guerrero Á, Riveiro M, Poca M-A, et al. Is Spreading Depolarization a Risk Factor for Late Epilepsy? A Prospective Study in Patients with Traumatic Brain Injury and Malignant Ischemic Stroke Undergoing Decompressive Craniectomy. Neurocrit Care. 2021 Jun;34(3):876–88.

3. Sueiras M, Thonon V, Santamarina E, Sánchez-Guerrero Á, Poca MA, Quintana M, et al. Cortical Spreading Depression Phenomena Are Frequent in Ischemic and Traumatic Penumbra: A Prospective Study in Patients With Traumatic Brain Injury and Large Hemispheric Ischemic Stroke. J Clin Neurophysiol Off Publ Am Electroencephalogr Soc. 2021 Jan;38(1):47–55.

4. Khellaf A, Garcia NM, Tajsic T, Alam A, Stovell MG, Killen MJ, et al. Focally administered succinate improves cerebral metabolism in traumatic brain injury patients with mitochondrial dysfunction. J Cereb blood flow Metab Off J Int Soc Cereb Blood Flow Metab. 2022 Jan;42(1):39–55.

5. Dreier JP, Major S, Foreman B, Winkler MKL, Kang E-J, Milakara D, et al. Terminal spreading depolarization and electrical silence in death of human cerebral cortex. Ann Neurol. 2018 Feb;83(2):295–310.
